# The Uptake of Integrated Perinatal Prevention of Mother-to-Child HIV Transmission Programs in Low- and Middle-Income Countries: A Systematic Review

**DOI:** 10.1371/journal.pone.0056550

**Published:** 2013-03-06

**Authors:** Lorainne Tudor Car, Serena Brusamento, Hoda Elmoniry, Michelle H. M. M. T. van Velthoven, Utz J. Pape, Vivian Welch, Peter Tugwell, Azeem Majeed, Igor Rudan, Josip Car, Rifat Atun

**Affiliations:** 1 Department of Primary Care and Public Health, School of Public Health, Imperial College London, London, United Kingdom; 2 Global eHealth Unit, Department of Primary Care and Public Health, School of Public Health, Imperial College London, London, United Kingdom; 3 Centre for Global Health, University of Ottawa, Ottawa, Canada; 4 Department of Medicine, University of Ottawa, Ottawa, Canada; 5 Centre for Population Health Sciences, University of Edinburgh Medical School, Edinburgh, United Kingdom; 6 Imperial College Business School, Imperial College London, London, United Kindom; Africa Centre for Health and Population Studies - University of KwaZulu-Natal, South Africa

## Abstract

**Background:**

The objective of this review was to assess the uptake of WHO recommended integrated perinatal prevention of mother-to-child transmission (PMTCT) of HIV interventions in low- and middle-income countries.

**Methods and Findings:**

We searched 21 databases for observational studies presenting uptake of integrated PMTCT programs in low- and middle-income countries. Forty-one studies on programs implemented between 1997 and 2006, met inclusion criteria. The proportion of women attending antenatal care who were counseled and who were tested was high; 96% (range 30–100%) and 81% (range 26–100%), respectively. However, the overall median proportion of HIV positive women provided with antiretroviral prophylaxis in antenatal care and attending labor ward was 55% (range 22–99%) and 60% (range 19–100%), respectively. The proportion of women with unknown HIV status, tested for HIV at labor ward was 70%. Overall, 79% (range 44–100%) of infants were tested for HIV and 11% (range 3–18%) of them were HIV positive. We designed two PMTCT cascades using studies with outcomes for all perinatal PMTCT interventions which showed that an estimated 22% of all HIV positive women attending antenatal care and 11% of all HIV positive women delivering at labor ward were not notified about their HIV status and did not participate in PMTCT program. Only 17% of HIV positive antenatal care attendees and their infants are known to have taken antiretroviral prophylaxis.

**Conclusion:**

The existing evidence provides information only about the initial PMTCT programs which were based on the old WHO PMTCT guidelines. The uptake of counseling and HIV testing among pregnant women attending antenatal care was high, but their retention in PMTCT programs was low. The majority of women in the included studies did not receive ARV prophylaxis in antenatal care; nor did they attend labor ward. More studies evaluating the uptake in current PMTCT programs are urgently needed.

## Introduction

In 2010, an estimated 16.8 million women and 3.4 million children were HIV positive [1]. Each day an estimated 1000 children under the age of 16 acquire HIV infection [1], over 90% of them due to mother-to-child transmission (MTCT) of HIV [1]. Transmission of HIV from mother to child can be reduced from 15–40% to 1% by effective prevention of mother to child transmission (PMTCT) of HIV programs [1].

When PMTCT programs were initially introduced in low- and middle-income countries, they were stand-alone programs, with gradual integration into maternal and newborn healthcare services [2], [3] as one of the key strategies to improve coverage, achieve universal access to PMTCT programs [4]–[8], and improve the quality of care delivered through better and synergistic use of scarce human and financial resources [7]. Implementing PMTCT programs integrated to routine healthcare services might help to reduce the stigma often experienced by HIV infected people [9], [10]. However, many women in low- and middle-income countries do not attend maternal and child healthcare services [11]. Integrated or not, PMTCT services experience loss-to follow-up at each step of the program delivery: from the first contact, through counseling, HIV testing, collecting results, receiving antiretroviral therapy (ART) or prophylaxis, safe delivery practices, infant feeding recommendations up to postnatal follow-up; thereby reducing program effectiveness.

The uptake of PMTCT program is also influenced by the type of interventions implemented. The World Health Organization (WHO) has recommended the use of opt-out (i.e. offering testing routinely to all women entering care with an option to “opt-out” of testing), rapid HIV testing with return of results on the same day [12], and the use of virological tests in infants [13]. Virological test can be used earlier than an antibody test to identify HIV infections in infants since maternal HIV antibodies transferred passively during pregnancy can persist for as long as 18 months in children born to HIV-infected mothers and the interpretation of positive HIV antibody test results is more difficult in children below this age [14], [15]. The aim of the WHO recommended HIV testing methods is to increase the number of women and children tested for HIV and knowing their serostatus as well as to improve access to HIV treatment.

In 2001, at the United Nations General Assembly Special Session (UNGASS), a goal was set to reduce the proportion of HIV infected infants by 50% by the year 2010 [16]. In order to achieve this target, an estimated 80% of pregnant women and their infants would need to have access to essential prevention, treatment and care [17]. Despite substantial international efforts, the progress on 4 th and 5 th Millennium Development Goals has been lagging behind the target [18], [19].

Low coverage of PMTCT programs prompted the UN Secretary General and key UN agencies to call for increased domestic and international financing to address health of women and children to reach the 4 th and 5 th Millennium Development Goals and to expand the PMTCT coverage [20]. Furthermore, the UN Secretary General, G8 countries, and the Global Fund to Fight AIDS, Tuberculosis and Malaria, in collaboration with its key partners the Joint United Nations Program on HIV/AIDS (UNAIDS), WHO, the United Nations Children's Fund (UNICEF), the United Nations Population Fund (UNFPA) and the Children’s Investment Fund Foundation (CIFF) have committed to further develop and improve the quality and effectiveness of PMTCT services in low- and middle-income countries [20], [21]. Integration of PMTCT with other healthcare services is a crucial component of this initiative [4], [7], [8], [13], [22].

Given the unacceptably large number of children affected by HIV at birth [1], poor PMTCT coverage [11], the potential impact of successful PMTCT programs and the international commitment to the improvement of these services, it is important to assess the effectiveness of integration of PMTCT services with other health services, such as maternal, newborn, and child health services. While several literature overviews explore integration of individual PMTCT interventions, the evidence on the uptake of these programs has not been systematically assessed [23]–[26]. The objective of this systematic review is to assess the uptake of integrated PMTCT programs in low- and middle-income countries.

## Methods

### Definition

This review focuses on the integration of the third prong of PMTCT interventions (Text S1), i.e. perinatal PMTCT interventions, which consist of HIV testing and counseling, provision of ART or antiretroviral (ARV) prophylaxis to women, safe delivery practices, infant feeding counseling, provision of ARV prophylaxis and HIV testing to the infant [27],[28]. The PMTCT guidelines have changed several times since the beginning of PMTCT programs. This systematic review aims to present the overall uptake of PMTCT interventions and includes studies that implemented or followed different versions of guidelines over the study time period.

We considered eligible studies on integration of PMTCT with antenatal care, labor ward care, postnatal/follow-up care, child healthcare services (e.g. immunization), nutrition services, voluntary counseling and testing (VCT) centers, HIV clinics, sexually transmitted infection (STI) clinics, reproductive health services and out-patient clinics. Given that PMTCT interventions in many settings are integrated with maternal and child health services, we developed a framework describing the integration of PMTCT program with antenatal care, labor ward and postnatal care (Table S1).There is no internationally agreed definition of integrated care [29]–[32]. We defined the integration of PMTCT interventions as joining of service delivery of PMTCT programs with other health care services, either at a single point of access (unified, full) or through referrals [33].

### Inclusion and exclusion criteria

We included observational studies performed in low- and middle-income countries as defined by the World Bank [34]. Studies had to focus on pregnant women, women in labor or in postpartum and infants with unknown HIV serostatus at enrolment in an integrated PMTCT program.

The primary outcome was the uptake of PMTCT programs, i.e. the proportion of women and children receiving individual PMTCT interventions (Text S2). In addition, we collected other relevant outcomes such as the proportion of HIV negative infants born to HIV positive women, costs, the impact on human resources, the quality of care, stigma, inequalities in access, barriers to implementation and sustainability of integrated programs and the impact on attendance (Text S2). We also included information about the follow-up i.e. provision of ART and co-trimoxazole to HIV positive women and HIV positive infants after the delivery. Exclusion criteria are listed in Text S3.

### Search strategy

We performed a comprehensive search to identify all relevant research regardless of the publication status, in any language with an English abstract. The search was conducted from 1990 (time of the first implementation of PMTCT programs) to August 2010. We used a highly sensitive search strategy developed in collaboration with the Cochrane HIV/AIDS Review Group. The search strategy did not entail search terms related to integrated care because in some studies “integration” could have been omitted or be apparent only from the description of service. We examined description of service delivery in each study to decide if it fits our definition of healthcare service integration.

### Electronic searches

We searched the following electronic databases: MEDLINE, EMBASE, the Cochrane Library, WHO The Global Health Library, Global Health CAB abstracts, CINAHL, POPLINE, PsychINFO, Sociological Abstracts, ERIC, the NLM Gateway system, and the Controlled-trials register and the WHO International Clinical Trials Registry for ongoing trials on PMTCT. We also searched several grey literature sources: New York Academy of Medicine Grey Literature Collection, Google Scholar, Open SIGLE, British Library Catalogue, AEGIS and ProQuest Dissertation & Theses Database (Text S4). We contacted authors of conference proceedings for additional information.

### Screening process and data extraction

Two review authors independently performed selection of studies and data extraction. Disagreements between authors were resolved by discussion, consensus and consultation with a third review author. We screened titles and abstracts and then full-texts for eligibility. Data from the included studies were extracted using a standardized data extraction sheet (Table S2).

### Data analysis

We analyzed the data presented in the studies and performed narrative synthesis. The included studies used different denominators to present the proportion of participants receiving perinatal PMTCT intervention which we recalculated and standardized (Text S5). We separately analyzed the studies which described the implementation of the PMTCT program in antenatal care and at labor ward level. For pregnant women enrolled at antenatal care we separately described the proportion of women receiving ARV prophylaxis in antenatal care, the proportion receiving it at labor ward and the total proportion of women who received ARV prophylaxis at delivery (consisting of women who received ARV prophylaxis in antenatal care and self-reported that they have taken it during home delivery and women who took the prophylaxis at the labor ward).

Statistical analysis was carried out based on non-parametric tests since distributional assumptions were not satisfied. The Wilcoxon rank sum test was applied to perform all comparisons between two groups [35]. Differences in outcome for the three World Bank income groups, namely low-, lower-middle, upper-middle income countries were tested by an extended rank sum test developed by Cuzick [36]. We used a rank sum trend test, which is a natural extension of the Wilcoxon rank sum test for two groups, as a non-parametric statistic to consider the trend between more than two groups.

We designed two cascades with the data from the studies reporting on the uptake of each step of perinatal PMTCT programs [37], [38]. The first cascade presents integration of PMTCT interventions in antenatal care and the second cascade illustrates the integration of PMTCT interventions at labor ward. Both cascades pool the studies weighted by the number of participating women. The fractions are relative to the number of women attending antenatal care in the first cascade and the number of women eligible for HIV test at labor ward in the second cascade. The first cascade assumes that the HIV prevalence is equal among women who accepted and those who declined testing. This assumption is probably conservative as women might decline testing since they feel likely to be positive. Hence, the real number of HIV positive among women who declined testing is probably higher than our estimate. However, we imply that selection into testing did not result in a higher number of positive HIV tests, which could arguably be the case if once tested women refrain from a repeated test as they correctly assume that they are still negative. We made the same assumption for the second cascade to estimate the proportion of unidentified HIV positive women.

## Results

### Study characteristics

**Figure 1:**
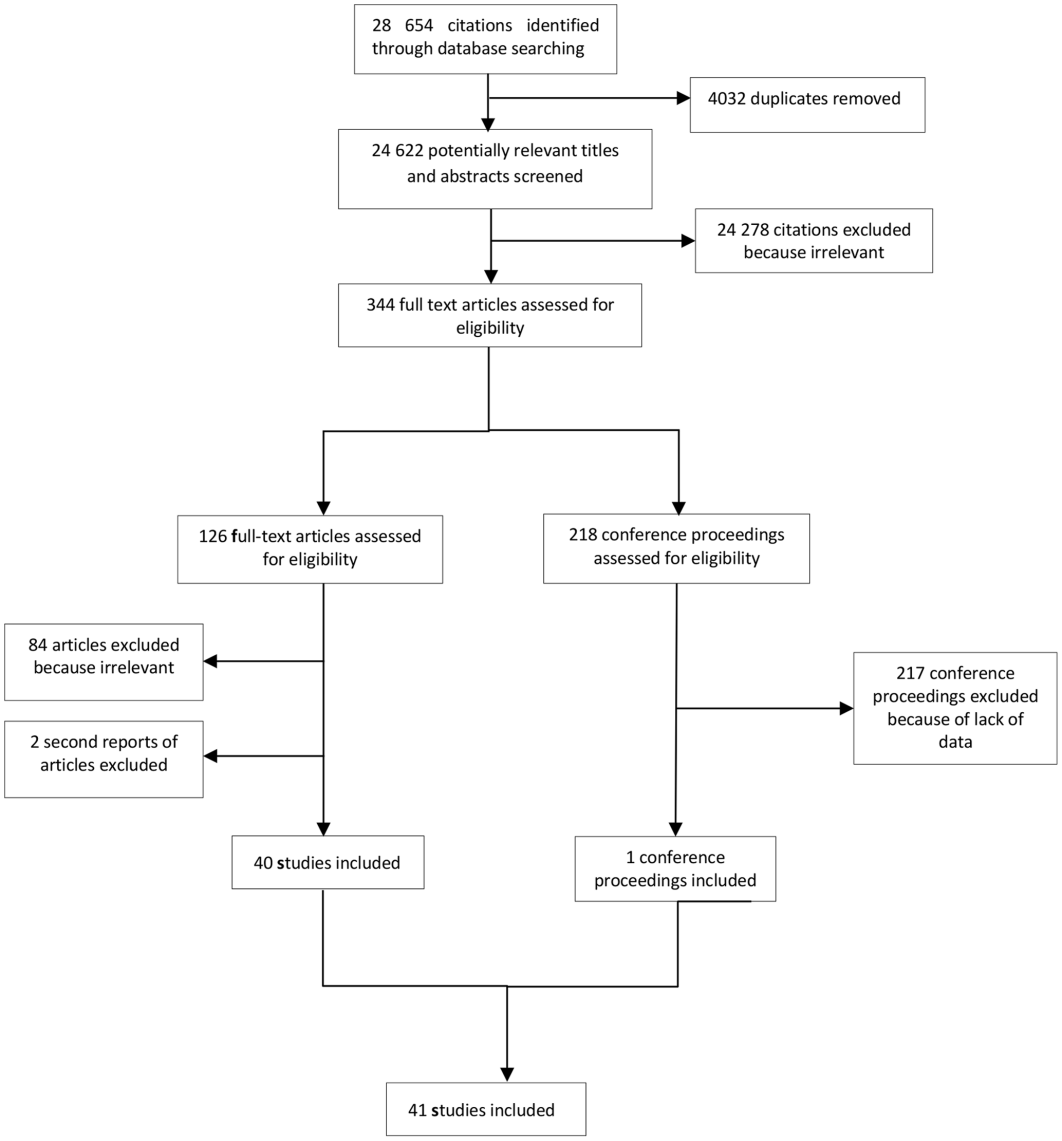
PRISMA Flow diagram.

Our search strategy identified 28 654 references (Figure 1 and Table S3). One hundred and twenty one articles and 218 relevant conference abstracts passed the first screening of titles and abstracts. After full text assessment, we excluded 86 articles (Table S4). We contacted authors of relevant conference abstracts for further information resulting in one included abstract. Forty one studies met inclusion criteria (Table 1 and Figure 1). We found two second reports: a French version of an English article [39], and a brief additional description of a program already reported in one of the included studies [40]. Except one article in Spanish [41], all the included studies were in English.

**Table 1:** Characteristics of included observational studies.

Author/Year/Location/Study period	Participants/Sample size (n)/Setting	Settings	Program Integration	Opt in vs opt out/Type of HIV test/ARV regimen (Woman/Infant)	Primary outcomes reported
Abdullah et al,[42] 2001 RSA Jan-99–Dec-00	Women ANC/ Attendees: 14 139/ Urban	PMTCT integration: across ANC, LW, PNC/ ANC and LW at primary healthcare level/ LW at hospital level	ANC: Testing women, Feeding counseling, Women prophylaxis/ LW: Women prophylaxis, Feeding counseling/ PNC: Testing infants	Opt-in/ Women: ELISA test later substitute with rapid test; Infants: antibody test/ Women: AZT; Infants: -	ANC: Percentage of women who received information on PMTCT; Percentage of women tested; Percentage of women who received their test result; Percentage of HIV positive women who received their test result; Percentage of women receiving ARV prophylaxis/ LW: Percentage of women receiving ARV prophylaxis
Behets et al, [50] 2009 DRC Jun-03 –Jul-05	Women ANC/ Attendees: 45 262/ Urban	PMTCT Integration: ANC, LW/ LW at hospital level	ANC: Testing women, Women prophylaxis/ LW: Women prophylaxis, Infant prophylaxis	Opt-in/ Women: unspecified; Infants: - / Women: sd-NVP; Infants: sd-NVP	ANC: Percentage of women who received their test result; Percentage of HIV positive women who received their test result; Percentage of women receiving ARV prophylaxis/ LW: Percentage of women receiving ARV prophylaxis/ PNC: Percentage of infants receiving ARV prophylaxis
Bharucha et al,[72] 2005 India Apr-01–Mar-02	Women LW/ Eligible: 1308/ Urban	PMTCT integration: LW/ LW at hospital level	LW: Testing women, Women prophylaxis	Opt-in/ Women: rapid test (+ confirmatory test); Infants: - / Women: sd-NVP or AZT during delivery; Infants: -	LW: Percentage of women who received information on PMTCT, Percentage of women tested, Percentage of women receiving ARV prophylaxis
Deschamps et al,[67] 2009 Haiti Jun-05- Jun-05	Women ANC/ HIV tested:5270/ Urban	PMTCT integration: ANC, PNC	ANC: Testing women, Women ART, Women prophylaxis/ LW: Women prophylaxis/ PNC: Feeding counseling, Testing infants, infant prophylaxis	Opt-in/ Women: rapid test; Infants: PCR within the first 6 months of life/ Women: sd-NVP or AZT orally during delivery; Infants : AZT for 1 week	ANC: Percentage of women tested, Percentage of women receiving ARV prophylaxis
Doherty et al,[67] 2005 RSA Jan-02 – Dec-02	Women ANC/ Attendees: 84 406/ Urban and rural	PMTCT integration: across ANC, LW, PNC/ ANC at primary healthcare level/ LW at hospital level	ANC: Testing women, Feeding counseling, Women prophylaxis/ LW: Infant prophylaxis/ PNC: Testing infants	-/ Women: rapid test; Infants: antibody test/ Women: sd-NVP; Infants: sd-NVP	ANC: Percentage of women who received information on PMTCT, Percentage of women tested, Percentage of women who received their test result, Percentage of women receiving ARV prophylaxis/ PNC: Percentage of infants receiving ARV prophylaxis, Percentage of infants tested, Percentage of exposed infants who resulted HIV negative
Ekouevi et al,[43] 2004 Côte d'Ivoire May- 00- Oct-02	Women ANC/ Counseled on PMTCT: 14 067/ Rural	PMTCT integration: ANC, LW/ ANC at primary healthcare level/ LW at hospital level	ANC: Testing women, Feeding counseling, Women prophylaxis/ LW: Women prophylaxis, Infant prophylaxis	Opt-in/ Women: rapid test (+ confirmatory test); Infants: -/ Women AZT from 36weeks plus sd-NVP; Infants: -	ANC: Percentage of women who received their test result, Percentage of HIV positive women who received their test result, Percentage of women receiving ARV prophylaxis
Garcia et al,[41] 2005 Colombia Mar-03- Mar-05	Women ANC/ HIV tested: 200 853/ Urban and Rural	PMTCT integration: across ANC, LW, PNC/ ANC at primary healthcare level/ LW at hospital level	ANC: Testing women/ LW: Women prophylaxis, Safe delivery, Infant prophylaxis/ PNC: Testing infants	Opt-in/ Women: ELISA and Western blot; Infants: Viral load within first 6 months of life/ Women: AZT-3TC/ AZT-3TC-NVP during pregnancy, AZT / sd-NVP/AZT+sd-NVP at delivery; Infants: AZT+3TC for 6 weeks	ANC: Percentage of women receiving ARV prophylaxis/ LW: Percentage of women receiving ARV prophylaxis, Percentage of women having elective caesarean section/ PNC: Percentage of infants receiving ARVs prophylaxis, Percentage of infants tested, Percentage of exposed infants who resulted HIV negative
Geddes et al,[66] 2008 RSA Mar-04 – Aug-05	Women ANC/ Attendees: 2624/ Urban	PMTCT integration: across ANC, LW, PNC/ ANC and LW at hospital level	ANC: Testing women, Feeding counseling, Women ART, Women prophylaxis/ LW: Women prophylaxis, Safe delivery, Infant prophylaxis/ PNC: Testing infants	Opt-in/ Women: rapid test; Infants: PCR within the first 6 months of life/ Women: AZT during pregnancy, sd-NVP/ AZT+sd-NVP at delivery or HAART for life; Infants: sd-NVP/ AZT+sd-NVP	ANC: Percentage of women who received information on PMTCT, Percentage of women tested, Percentage of women who received their test result, Percentage of women receiving ARV prophylaxis/ LW: Percentage of women having elective caesarean section/ PNC: Percentage of infants receiving ARVs prophylaxis, Percentage of infants tested, Percentage of exposed infants who resulted HIV negative
Hillis et al,[73] 2007 Russia Apr-04 – Oct-04	Women LW/ Eligible for HIV test: 709/ Urban	PMTCT integration: LW	LW: Testing women, Women prophylaxis, Infant prophylaxis	-/ Women: EIA and Western blot; Infants: - / Women: sd-NVP; Infants: sd-NVP	LW: Percentage of women who received information on PMTCT, Percentage of women tested, Percentage of women receiving ARV prophylaxis
Homsy et al,[52] 2006 Uganda Dec-04 – Sept-05	Women ANC/ Attendees: 3765/ Rural	PMTCT integration: across ANC, LW, PNC/ ANC and LW at hospital level	ANC: Testing women, Women ART (referral), Feeding counseling, Women prophylaxis/ LW: Testing women, Feeding counseling, Women prophylaxis, Infant prophylaxis/ PNC: Testing infants	Opt-out/ Women: rapid test; Infants: -/ Women: sd-NVP; Infants: sd-NVP	ANC: Percentage of women who received information on PMTCT, Percentage of women tested, Percentage of women receiving ARV prophylaxis/ LW: Percentage of women who received information on PMTCT, Percentage of women tested, Percentage of women receiving ARV prophylaxis/ PNC: Percentage of Infants receiving ARV prophylaxis
Kanshana et al,[74] 2000 Thailand Jul-98 - Jun-99	Women ANC/ Attendees: 60 403/ Urban and Rural	PMTCT integration: across ANC, LW, PNC/ LW at hospital level	ANC: Testing women, Feeding counseling, Women prophylaxis/ LW: Women prophylaxis/ PNC: Testing infants	Opt-in/ Women: rapid test (+ confirmatory test); Infants: antibody test/ Women: AZT from 36 weeks and delivery; Infants: -	ANC: Percentage of women who received information on PMTCT, Percentage of women tested/ LW: Percentage of women receiving ARV prophylaxis
Karcher et al,[53] 2006 Tanzania Uganda Mar-02 – Dec-04	Women ANC/ Tanzania Attendees: 22 664/ Uganda Attendees: 29 520/ Urban and rural	PMTCT integration: across ANC, LW, PNC/ ANC at primary healthcare level/ LW at hospital level	ANC: Testing women, Women prophylaxis/ LW: Women prophylaxis, Infant prophylaxis/ PNC: Feeding counseling	-/ Women: rapid test; Infants: - / Women: sd-NVP; Infants: sd-NVP	ANC: Percentage of women who received information on PMTCT, Percentage of women tested, Percentage of women receiving ARV prophylaxis/ LW: Percentage of women receiving ARV prophylaxis/ PNC: Percentage of Infants receiving ARVs prophylaxis
Kasenga et al,[54] 2007 Malawi Jan-05 – Jun-05	Women ANC/ Attendees: 719/ Rural	PMTCT integration: across ANC, LW, PNC/ ANC and LW at hospital level	ANC: Testing women, Women prophylaxis/ LW: Infant prophylaxis/ PNC: Feeding counseling, Testing infants	Opt-out/ Women: unspecified; Infants: antibody test/ Women: sd-NVP; Infants: sd-NVP	ANC: Percentage of women who received information on PMTCT, Percentage of women tested, Percentage of women receiving ARV prophylaxis/ LW: Percentage of women receiving ARV prophylaxis/ PNC: Percentage of infants receiving ARV prophylaxis
Kirere et al,[55] 2008 DRC Dec-02 – Dec-04	Women ANC/ HIV tested: 5016/ Rural	PMTCT integration: ANC, LW/ ANC and LW at hospital level	ANC: Testing women/ LW: Women prophylaxis, Infant prophylaxis	-/ Women: rapid test; Infants: - / Women: sd-NVP; Infants: sd-NVP	LW: Percentage of women receiving ARV prophylaxis/ PNC: Percentage of infants receiving ARV prophylaxis
Kissin et al,[75] 2008 Russia Apr-04 – Apr-05	Women LW/ Eligible for HIV test: 4117/ Urban	PMTCT integration: across ANC, LW, PNC/ LW at hospital level	ANC: Testing women/ LW: Testing women, Feeding counseling, Women prophylaxis, Infant prophylaxis, Women ART (referral), Testing infants/ PNC: Testing infants	Opt-out/ Women: rapid test (+ confirmatory test); Infants; PCR within the first six months of life/ Women: sd-NVP; Infants: sd-NVP	LW: Percentage of women who received information on PMTCT, Percentage of women tested, Percentage of women receiving ARV prophylaxis/ PNC: Percentage of infants receiving ARV prophylaxis, Percentage of infants tested, Percentage of exposed infants who resulted HIV negative
Kuam et al,[56] 2006 Cameroon Jan-03 – Dec-04	Women ANC/ Attendees: 432/ Urban	PMTCT integration: across ANC, LW, PNC/ ANC and LW at hospital level	ANC: Testing women, Women prophylaxis/ LW: Safe delivery, Feeding counseling, Women prophylaxis, Infant prophylaxis/ PNC: Feeding counseling, Testing infants	-/Women: rapid test (+ confirmatory test); Infants: Viral load/ Women: sd-NVP; Infants: sd-NVP	ANC: Percentage of women who received information on PMTCT, Percentage of women tested, Percentage of women who received their test result, Percentage of HIV positive women who received their test result, Percentage of women receiving ARV prophylaxis/ LW: Percentage of women having elective caesarean section/ PNC: Percentage of infants receiving ARV prophylaxis, Percentage of infants tested, Percentage of exposed infants who resulted HIV negative
Le et al,[76] 2008 Vietnam Jan-00 – Oct-02	Women ANC; Women LW/ Total attendees: 215 683/ Urban	PMTCT integration: ANC, LW/ LW at hospital level	ANC: Testing women, Women prophylaxis/ LW: Testing women, Feeding counseling, Women prophylaxis, Infant prophylaxis	Mandatory/ Women: EIA (+ confirmatory test); Infants: - / Women: sd-NVP or AZT at delivery; Infants: sd-NVP/AZT +sd-NVP	LW: Percentage of women receiving ARV prophylaxis/ PNC: Percentage of infants receiving ARVs prophylaxis
Magoni et al,[57] 2007 Uganda Jan-00 – Dec-04	Women ANC/ Attendees: 26 556/ Urban	PMTCT integration: ANC, PNC/ ANC at hospital level	ANC: Testing women, Feeding counseling, Women prophylaxis, Testing infants/ PNC: Testing infants	-/Women: rapid test; Infants: antibody test/ Women: AZT from 36 weeks, AZT or sd-NVP at delivery; Infants: -	ANC: Percentage of women who received information on PMTCT, Percentage of women tested, Percentage of women receiving ARV prophylaxis/ PNC: Percentage of infants tested, Percentage of exposed infants who resulted HIV negative
Malyuta et al,[81] 2006 Ukraine Jan-02 – Dec-02	Women ANC/ Attendees: 408 354/ Urban	PMTCT integration: across ANC (HIV Clinic), LW, PNC/ ANC at primary healthcare level/ LW at hospital level	ANC: Testing women, Women prophylaxis/ LW: Testing women, Women prophylaxis, Infant prophylaxis/ PNC: Testing infants/ AIDS center: Feeding counseling, Testing infants	Opt-in/Women: ELISA in ANC, rapid test at LW; Infants: antibody test/ Women: AZT from 36 weeks, AZT or sd-NVP at delivery; Infants: sd-NVP	ANC: Percentage of women tested, Percentage of women receiving ARV prophylaxis/ LW: Percentage of women receiving ARV prophylaxis
Manzi et al,[58] 2005 Malawi Mar-02 – Sep-03	Women ANC/ Attendees: 3136/-	PMTCT integration: across ANC, LW, PNC/ ANC and LW at hospital level	ANC: Testing women, Safe delivery, Feeding counseling, Women prophylaxis/ LW: Safe delivery, Feeding counseling, Women prophylaxis, Infant prophylaxis/ PNC: Testing infants	Opt-out/ Women: rapid test; Infants: antibody test/ Women: sd-NVP; Infants: sd-NVP	ANC: Percentage of women who received information on PMTCT, Percentage of women tested, Percentage of women who received their test result, Percentage of HIV positive women who received their test result, Percentage of women receiving ARV prophylaxis/ LW: Percentage of women receiving ARV prophylaxis/ PNC: Percentage of infants receiving ARVs prophylaxis
Miranda et al,[68] 2005 Brazil 1997 – 2001	Women ANC/ Attendees: 25 224/ Urban	PMTCT integration: across ANC, LW, PNC/ ANC at primary healthcare level/ LW at hospital level	ANC: Testing women, Women prophylaxis/ LW: Testing women, Feeding counseling, Women prophylaxis, Infant prophylaxis/ PNC: Testing infants	Opt-out/ Women: ELISA (+ confirmatory test); Infants: PCR within the first six months of life/ Women: AZT from 14 weeks and delivery; Infants: AZT for 6 weeks	ANC: Percentage of women receiving ARV prophylaxis/ LW: Percentage of women receiving ARV prophylaxis, Percentage of women having elective caesarean section/ PNC: Percentage of infants receiving ARVs prophylaxis, Percentage of infants tested, Percentage of exposed infants who resulted HIV negative
Moth et al,[59] 2005 Kenya Feb-03 – Mar-03	Women ANC/ Attendees: 1268/ Urban	PMTCT integration: ANC, LW/ ANC and LW at hospital level	ANC: Testing women/ LW: Women prophylaxis, Infant prophylaxis	-/ Women: unspecified; Infants: - / Women: sd-NVP; Infants: sd-NVP	ANC: Percentage of women who received information on PMTCT, Percentage of women tested/ LW: Percentage of women receiving ARV prophylaxis/ PNC: Percentage of infants receiving ARVs prophylaxis
Msellati et al,[44] 2001 Côte d'Ivoire Oct-98 - Apr- 99	Women ANC/ Attendees: 4309/ Urban	PMTCT integration: ANC, LW/ ANC at primary healthcare level/ LW at hospital level	ANC: Testing women, Feeding counseling, Women prophylaxis/ LW: Women prophylaxis	Opt-in/ Women: ELISA test; Infants: - / Women: AZT from 36 weeks and delivery; Infants: -	ANC: Percentage of women who received information on PMTCT, Percentage of women tested, Percentage of women who received their test result, Percentage of HIV positive women who received their test result, Percentage of women receiving ARV prophylaxis/ LW: Percentage of women receiving ARV prophylaxis
Nagdeo et al,[77] 2007 India Feb-03 – Jun-06	Women ANC/ Attendees: 7897/ Rural	PMTCT integration: ANC, LW/ ANC at primary healthcare level/ LW at hospital level	ANC: Testing women/ LW: Women prophylaxis, Infant prophylaxis	-/ Women: unspecified; Infants: - / Women: sd-NVP; Infants: sd-NVP	LW: Percentage of women receiving ARV prophylaxis/ PNC: Percentage of infants receiving ARV prophylaxis
Onah et al,[45] 2008 Nigeria Mar-05 - Sept-05	Women ANC/ Attendees: 635/ Urban	PMTCT integration: ANC, LW/ ANC and LW at hospital level	ANC: Testing women, Feeding counseling, Women prophylaxis, Safe delivery/ LW: Women prophylaxis, Safe delivery, Infant prophylaxis, Feeding counseling	Opt-out/ Women: ELISA and Western blot; Infants: - / Women: sd-NVP; Infants: sd-NVP	ANC: Percentage of women who received information on PMTCT, Percentage of women tested, Percentage of women who received their test result, Percentage of HIV positive women who received their test result/ LW: Percentage of women receiving ARV prophylaxis, Percentage of women having elective caesarean section/ PNC: Percentage of infants receiving ARVs prophylaxis
Pai et al,[78] 2008 India Jan-06 – Sep-06	Women LW/ Eligible for HIV test: 1252/ Rural	PMTCT integration: LW, PNC/ LW at hospital level	LW: Testing women, Feeding counseling, Women prophylaxis, Safe delivery, Infant prophylaxis/ PNC: Testing infants, Feeding counseling	-/ Women: rapid test (+confirmatory test); Infants: PCR within the first 6 months of life/ Women: AZT-3TC-sd-NVP during delivery plus AZT-3TC 1 week after delivery; Infants: sd-NVP + AZT for 1 week	LW: Percentage of women who received information on PMTCT, Percentage of women tested, Percentage of women receiving ARV prophylaxis/ PNC: Percentage of infants receiving ARVs prophylaxis, Percentage of infants tested, Percentage of exposed infants who resulted HIV negative
Parameshwari et al,[79] 2009 India Oct-02 – Dec-07	Women ANC/ Attendees: 7866/ Rural	PMTCT integration: across ANC, LW, PNC/ ANC and LW at hospital level	ANC: Testing women, Feeding counseling, Women prophylaxis/ LW: Women prophylaxis, Infant prophylaxis, PNC, Testing infants	-/ Women: rapid test (+ confirmatory test); Infants: PCR within the first 6 months of life/ Women: sd-NVP; Infants: sd-NVP	ANC: Percentage of women who received information on PMTCT, Percentage of women tested, Percentage of women receiving ARV prophylaxis/ PNC: Percentage of infants receiving ARV prophylaxis, Percentage of infants tested, Percentage of exposed infants who resulted HIV negative
Perez et al,[46], [94] 2004 Zimbabwe Aug-01- Feb-03	Women ANC/ Attendees: 2470/ Rural	PMTCT integration: across ANC, LW, PNC/ ANC and LW at hospital level	ANC: Testing women, Feeding counseling/ LW: Women prophylaxis, Infant prophylaxis/ PNC: Feeding counseling	Opt-in/ Women: rapid test; Infants: - /Women: sd-NVP; Infants: sd-NVP	ANC: Percentage of women who received information on PMTCT, Percentage of women tested, Percentage of women who received their test result, Percentage of HIV positive women who received their test result, Percentage of women receiving ARV prophylaxis/ LW: Percentage of women receiving ARV prophylaxis/ PNC: Percentage of infants receiving ARVs prophylaxis
Rose et al,[60] 2005 RSA Jan-03 – Dec-05	Women LW/ Eligible for HIV test: 7500/ Urban	PMTCT integration: LW (postpartum)/ LW at hospital level	LW: Testing women, Infant prophylaxis, Feeding counseling	-/ Women: rapid test (+ confirmatory test); Infants: - / Women: - ; Infants: sd-NVP	LW: Percentage of women who received information on PMTCT, Percentage of women tested/ PNC: Percentage of infants receiving ARVs prophylaxis
Rutta et al,[47] 2008 Tanzania Oct-02 – Sep-04	Women ANC/ Attendees: 10 666/ Refugees camp	PMTCT integration: across ANC, LW, PNC/ ANC at primary healthcare level/ LW at hospital level	ANC: Testing women, Feeding counseling, Safe delivery, Women prophylaxis/ LW: Safe delivery, Women prophylaxis, Infant prophylaxis/ PNC: Testing infants	Opt- out/ Women: rapid test (+ confirmatory test); Infants: antibody test/ Women: sd-NVP; Infants: sd-NVP	ANC: Percentage of women who received information on PMTCT, Percentage of women tested, Percentage of women who received their test result, Percentage of HIV positive women who received their test result, Percentage of women receiving ARV prophylaxis/ LW: Percentage of women receiving ARV prophylaxis/ PNC: Percentage of infants receiving ARV prophylaxis, Percentage of infants tested, Percentage of exposed infants who resulted HIV negative
Saman et al,[80] 2002 Cambodia July-00 – Jun-01	Women ANC/ Attendees: 2079/ Urban	PMTCT integration: across ANC, LW, PNC/ ANC and LW at hospital level	ANC: Testing women, Feeding counseling/ LW: Testing women, Women prophylaxis, Infant prophylaxis, Feeding counseling/ PNC: Testing infants	-/ Women: rapid test (+ confirmatory test); Infants: PCR within the first 6 months of life/ Women: sd-NVP; Infants: sd-NVP	ANC: Percentage of women who received their test result/ LW: Percentage of women who received information on PMTCT, Percentage of women tested, Percentage of women receiving ARV prophylaxis/ PNC: Percentage of infants receiving ARVs prophylaxis
Saraceni et al,[69] 2000 Brazil Jun-97 – Jun-05	Women ANC/ Attendees: 202 950-	PMTCT integration: across ANC, LW, PNC/ LW at hospital level	ANC: Testing women, Feeding counseling, Women ART (referral), Women prophylaxis/ LW: Women prophylaxis, Safe delivery, Infant prophylaxis/ PNC: Feeding counseling, Testing infants	-/ Women: ELISA substituted with rapid test; Infants: antibody test/ Women: AZT during pregnancy and delivery; Infants: AZT	ANC: Percentage of women who received information on PMTCT, Percentage of women tested, Percentage of women receiving ARV prophylaxis/ LW: Percentage of women receiving ARV prophylaxis, Percentage of women having elective caesarean section/ PNC: Percentage of infants tested, Percentage of exposed infants who resulted HIV negative
Schumacher et al,[70] 2009 Guyana Jan-06 – Mar-06	Women LW/ HIV status unknown: 360	PMTCT integration: LW/ LW at hospital level	LW: Testing women, Women prophylaxis, Infant prophylaxis	-/ Women: unspecified; Infants: - / Women: sd-NVP, Infants: sd-NVP	LW: Percentage of women who received information on PMTCT, Percentage of women tested/ PNC: Percentage of infants receiving ARV prophylaxis
Shetty et al,[62] 2005 Zimbabwe July-99 - June-01	Women ANC/ Attendees: 6051/ Urban	PMTCT integration: ANC/ ANC at primary healthcare level	ANC: Testing women, Women prophylaxis	Opt-in/ Women: rapid test (+ confirmatory test); Infants: - / Women: AZT from 36 weeks; Infants: -	ANC: Percentage of women who received information on PMTCT, Percentage of women tested, Percentage of women who received their test result, Percentage of HIV positive women who received their test result, Percentage of women receiving ARV prophylaxis
Shetty et al,[61] 2008 Zimbabwe Oct-02 - Dec-04	Women ANC/ Attendees: 19 279/ Urban	PMTCT integration: across ANC, LW, PNC (Immunization clinic)/ ANC at primary healthcare level/ LW at hospital level	ANC: Testing women, Feeding counseling, Women prophylaxis/ LW: Women prophylaxis, Infant prophylaxis/ PNC: Testing infants	Opt-in/ Women: rapid test; Infants: antibody test/ Women: sd-NVP; Infants: sd-NVP	ANC: Percentage of women who received information on PMTCT, Percentage of women tested, Percentage of women who received their test result, Percentage of HIV positive women who received their test result, Percentage of women receiving ARV prophylaxis/ LW: Percentage of women receiving ARV prophylaxis/ PNC: Percentage of infants receiving ARVs prophylaxis
Stringer et al,[48] 2003 Zambia Nov-01- Oct-02	Women ANC/ Counseled on PMTCT: 17 263/ Urban	PMTCT integration: across ANC, LW, PNC/ LW at hospital level	ANC: Testing women, Women prophylaxis, Feeding counseling/ LW: Women prophylaxis, Infant prophylaxis/ PNC: Testing infants	Opt-in/ Women: rapid test; Infants: antibody test/ Women: sd-NVP; Infants: sd-NVP	ANC: Percentage of women who received their test result, Percentage of women receiving ARV prophylaxis
Temmerman et al,[49] 2003 Kenya Apr- 01 - Apr- 02	Women ANC/ Attendees: 3660/ Urban	PMTCT integration: ANC, LW/ ANC and LW at hospital level	ANC: Testing women, Feeding counseling, Women prophylaxis, Infant prophylaxis/ LW: Women prophylaxis, Infant prophylaxis	Opt-in/ Women: rapid test; Infants: - / Women: sd-NVP; Infants: sd-NVP, provided at ANC to administer during home delivery	ANC: Percentage of women who received information on PMTCT, Percentage of women tested, Percentage of women who received their test result, Percentage of HIV positive women who received their test result, Percentage of women receiving ARV prophylaxis/ LW: Percentage of women receiving ARV prophylaxis
Torpey et al,[63] 2010 Zambia Jul-05 – Sep-08	Women ANC/ Counseled on PMTCT: 34 780/ Urban and Rural	PMTCT Integration: across ANC, LW, PNC (Immunization clinic)/ANC at primary healthcare level/ LW at hospital level	ANC: Testing women, Women prophylaxis	Opt-out/ Women: rapid test (+ confirmatory test); Infants: PCR within the first 6 months of life/ Women: sd-NVP; Infants: sd-NVP	ANC: Percentage of women who received their test result, Percentage of HIV positive women who received their test result, Percentage of women receiving ARV prophylaxis
Viani et al,[71] 2010 Mexico Mar- 03 - Dec-05	Women ANC/ 17 at ANC and 45 at LW/ Urban	PMTCT integration: across ANC, LW, PNC/ ANC and LW at hospital level	ANC: Testing women, Feeding counseling, Women prophylaxis/ LW: Testing women, Women prophylaxis, Safe delivery, Infant prophylaxis, Feeding counseling/ PNC: Testing infants	-/ Women: rapid test (+ confirmatory test); Infants: PCR within the first six months of life/ Women: AZT-3TC-NFV during pregnancy or AZT during delivery; Infants: AZT for 6 weeks	ANC: Percentage of women receiving ARV prophylaxis, Percentage of women having elective caesarean section/ PNC: Percentage of infants receiving ARV prophylaxis, Percentage of infants tested, Percentage of exposed infants who resulted HIV negative
Wanyu et al,[64] 2007 Cameroo Jul-02 – Jun-05	Women ANC/ Counseled on PMTCT: 2331/ Rural	PMTCT integration: across ANC, LW, PNC/ ANC at primary healthcare level/ LW at hospital level	ANC: Testing women, Feeding counseling/ LW: Women prophylaxis, Infant prophylaxis/ PNC: Testing infants	Opt-in/ Women: rapid test; Infants: antibody test/Women: sd-NVP; Infants: sd-NVP	ANC: Percentage of women who received their test result, Percentage of HIV positive women who received their test result/ LW: Percentage of women receiving ARV prophylaxis/ PNC: Percentage of infants receiving ARV prophylaxis, Percentage of infants tested, Percentage of exposed infants who resulted HIV negative
Welty et al,[65] 2005 Cameroon Feb-00 – Dec-04	Women ANC; Counseled on PMTCT: 68 635/ Urban and rural	PMTCT integration: across ANC, LW, PNC/ ANC at primary healthcare level/ LW at hospital level	ANC: Testing women, Feeding counseling, Women prophylaxis/ LW: Women prophylaxis, Infant prophylaxis/ PNC:Testing infants	-/ Women: rapid test; Infants: PCR within the first 6 months of life/ Women: sd-NVP; Infants: sd-NVP	ANC: Percentage of women who received their test result, Percentage of HIV positive women who received their test result/ PNC: Percentage of exposed infants who resulted HIV negative

Abbreviations: ANC, antenatal care; ART, antiretroviral therapy; ARV, antiretroviral; AZT, zidovudine; DRC, Democratic Republic of Congo; EIA, enzyme immunoassay; ELISA, enzyme-linked immunosorbent assay; HAART, highly active antiretroviral therapy; LW, labor ward; NVF, nelfinavir; PMTCT, prevention of mother to child transmission; PCR, polymerase chain reaction; PNC, postnatal care; RSA, Republic of South Africa; sd-NVP, single dose nevirapine;3TC, lamivudine.

Twenty five studies were conducted in Sub-Saharan Africa [42]–[66], six in Latin America-Caribbean [41], [67]–[71], nine in Asia [72]–[80], and one in Ukraine [81]. Of the 41 included studies, 17 described PMTCT programs in low-income [46]–[50], [52]–[55], [57]–[59], [61]–[63], [67], [80], 15 in lower-middle income [41], [43]–[45], [56], [64], [65], [70], [72], [74], [76]–[79], [81], and nine in upper-middle income countries [42], [51], [60], [66], [68], [69], [71], [73], [75]. The presented PMTCT programs were implemented at different time periods between years 1997 and 2006 (Figure S1). Twelve programs occurred in countries where HIV prevalence was between 5% and 10% (Table S5). The general HIV prevalence in 18 studies was less than 5% and more than 10% in 11 programs, all of which were implemented in Africa (Table S5). PMTCT interventions were mostly integrated with maternal and child health services, i.e. antenatal care, labor wards, and postnatal care. The infant follow up was integrated with immunization centers in two studies [61], [63]. In one study the PMTCT was integrated with HIV/AIDS treatment centers [81]. Participants, staff, setting and interventions from integrated PMTCT programs are presented in Texts S6 and S7 and Table S6.

### Outcomes

#### Primary outcomes

In 34 studies women were enrolled in integrated PMTCT program in antenatal care. In most of these studies (n = 28), women were followed from antenatal care until delivery [41]–[51], [53]–[59], [61]–[69], [71], [74]–[77], [79], [81]. In seven studies women were enrolled into the integrated PMTCT programs at labor ward [60], [70]–[73], [75], [78]. In two studies women entered the integrated program both at antenatal and labor ward care [80], [82].

#### Integration of perinatal PMTCT program with antenatal care

The main outcomes from the 34 studies which reported on the PMTCT program integrated in antenatal care are displayed in Table 2. We performed subgroup analyses to analyze the impact of different strategies on the coverage within studies focusing on antenatal care integration (Text S8).

**Table 2:** Uptake of integrated PMTCT programs in which women were enrolled in antenatal care.

World Bank Income Group	Low Income	Lower Middle Income	Upper Middle Income	ALL	*P*
	Median (range)n studies	Median (range) n studies	Median (range) n studies	Median (range) n studies	
**Percentage of ANC attendees who received information on PMTCT**	92% (30–98%) 12	100% (87–100%) 5	100% (100–100%) 4	96% (30–100%) 21	0.001^a^
**Percentage of ANC attendees HIV tested**	69% (26–95%) 12	89% (80–100%) 6	81% (56–100%) 4	81% (26–100%) 22	0.048^b^
**Percentage of HIV tested ANC attendees who received HIV test result**	89% (74–100%) 10	81% (67–100%) 6	99% (85–100%) 3	87% (67–100%) 19	0.680
**Percentage of HIV positive ANC attendees who received HIV test result**	93% (75–100%) 8	92% (60–100%) 6	96% (92–100%) 2	94% (60–100%) 16	0.957
**Percentage of HIV positive ANC attendees given ARV prophylaxis at ANC**	53% (33–99%) 14	40% (22–89%) 5	60% (44–97%) 6	55% (22–99%) 25	0.784
**Percentage of HIV positive ANC attendees given ARV prophylaxis at LW (on women who delivered at LW)**	70% (19–100%) 11	70% (14–91%) 6	76% (43–95%) 5	74% (14–100%) 22	0.767
**Percentage of HIV positive ANC attendees known to have taken ARV prophylaxis at delivery (self-reported and observed by nurses)**	38% (19–75%) 12	60% (14–69%) 5	72% (43–85%) 5	52% (14–85%) 22	0.099

Abbreviations: ANC, antenatal care; ARV, antiretroviral; LW, labor ward; PMTCT, prevention of mother to child transmission.

a RankSumTrend test *P* = 0.001.

b RankSumTrend test *P* = 0.136.

#### Integration of perinatal PMTCT program with labor ward care

The primary outcomes reported in the studies which focused on integration of PMTCT with labor ward are shown in Table 3 [52], [60], [70]–[73], [75], [78], [80]. In those studies pregnant women were eligible for HIV testing if they had unknown or undocumented HIV status at their entrance in labor ward. Additionally, in two studies from Russia women who had a previous negative HIV test performed before 34 weeks of pregnancy were also considered eligible for repeat testing [75].

**Table 3:** Uptake of integrated PMTCT programs in which women were enrolled at a labor ward.

World Bank income group	Low income	Lower middle income	Upper middle income	All	*P*	
	Median (range) n studies	Median (range) n studies	Median (range) n studies	Median (range) n studies		
**Percentage of women delivering at LW who received information on PMTCT**	69% (53–84%) 2	68% (59–100%) 3	100% (77–100%) 3	80% (53–100%) 8	0.306	
**Percentage of women delivering at LW who were HIV tested**	59% (45–72%) 2	68% (44–98%) 3	89% (51–95%) 3	70% (44–98%) 8	0.505	
**Percentage of HIV positive women delivering at LW given ARV prophylaxis**	100% (N/A) 1	100% (100–100%) 2	77% (48–84%) 3	92% (48–100%) 6	0.351	
**Percentage of infants of HIV positive women delivering at LW given ARV prophylaxis**	94% (89–100%) 2	88% (75–100%) 2	96% (85–98%) 3	96% (75–100%) 7	0.558	

Abbreviations: ARV, antiretroviral; LW, labor ward; PMTCT, prevention of mother to child transmission of HIV.

#### Integration of perinatal PMTCT program with labor ward care: safe delivery

Only nine studies had quantitative data on safe delivery practices [41], [45], [47], [56], [58], [66], [68], [69], [71]. In two studies performed in low-income countries caesarean section was not available [47], [58]. In the other seven studies the proportion of HIV positive pregnant women who underwent elective caesarean section varied from 17% to 63% (median 28%) [41], [45], [56], [68], [69], [71]. Five of these studies were performed in upper-middle income countries [41], [66], [68], [69], [71], [83] and two in lower-middle income countries [45], [56].

Fifteen studies provided extractable data on the proportion of women who entered into the PMTCT program in antenatal care and delivered at hospital [41], [45], [47], [54], [56]–[59], [61], [64], [66], [68], [69], [74], [77]. The median proportion was 60% (range 19–100%), and it significantly varied across the World Bank income groups: 44% (range 19–60%) in studies performed in low-income [47], [54], [58], [59], [61], 86% (range 43–100%) in lower-middle income [45], [56], [64], [74], [77], and 100% (range 89–100%) among the upper-middle income countries (*P*<0·0001) [41], [66], [69].

#### Integration of perinatal PMTCT program with postnatal/follow-up care

The outcomes from the studies reporting coverage of infant ARV prophylaxis and HIV testing are presented in Table 4. Within the 13 studies with data on infant testing [41], [47], [51], [56], [57], [64], [66], [68], [69], [71], [75], [78], [79], six used a rapid test at 12, 15 and 18 months [47], [51], [64], [68], [69], [71], while in seven a PCR test or viral load was used within the first six months of life [41], [56], [57], [66], [75], [78], [79]. The proportion of children tested at least once was not significantly different between those two groups of studies, 82% (range 48–100%) versus 76% (range 44–100%), P = 0.548.

**Table 4:** Uptake of PMTCT interventions by infants born to HIV positive women.

World Bank income group	Low income	Lower middle income	Upper middle income	All	*P*
	Median (range) n studies	Median (range) n studies	Median (range) n studies	Median (range) n studies	
**Percentage of infants of HIV positive women given ARV prophylaxis**	90% (24–100%) 12	100% (75–100%) 7	98% (85–100%) 7	97% (24–100%) 26	0.327
**Percentage of infants of HIV positive women HIV tested at least once**	67% (54–79%) 2	74% (44–100%) 4	82% (50–100%) 7	79% (44–100%) 13	0.548

Abbreviations: ARV, antiretroviral.

A study on integrated PMTCT program in Vietnam is described separately due to particularities of the program. In this study, HIV testing in antenatal care and at labor ward was mandatory and women had to pay for it. Given that women had to wait for the results of the tests for at least one week, most of the women who were tested at labor ward and their infants did not receive ARV treatment. The authors stated that 28.3% of the HIV positive women and 4.1% of their infants received ARV prophylaxis. The study did not report separate results for women tested in antenatal care and those tested at labor ward [76].

The proportion of women counseled on infant feeding was not explicitly stated in any of the included studies but most of them reported that women participating in PMTCT programs received infant feeding recommendation as a part of the counseling.

#### PMTCT cascades

**Figure 2:**
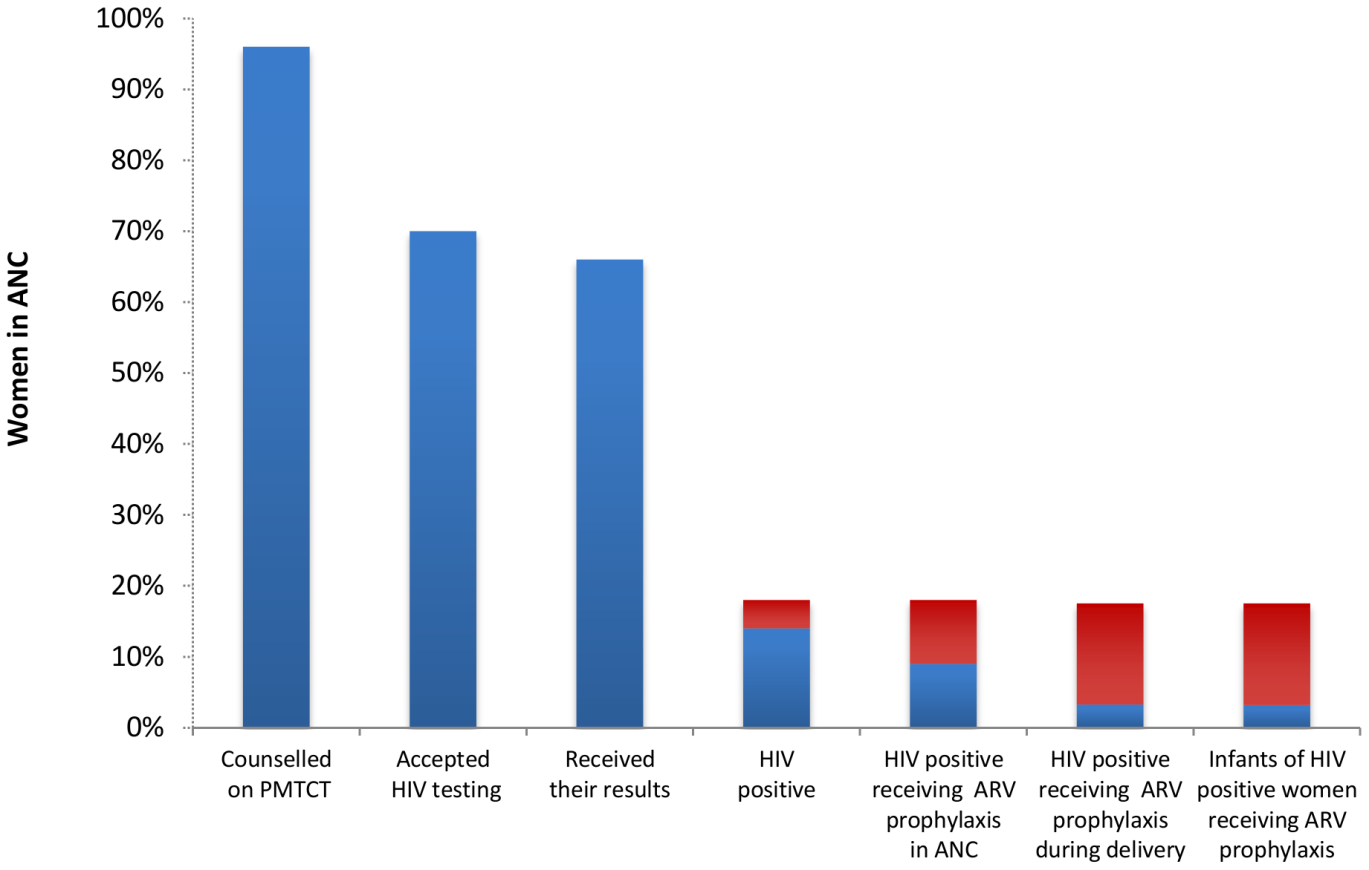
Cascade of integrated perinatal PMTCT program in antenatal care. Blue bars indicate proportion of women participating in PMTCT program while red bars show the estimated proportion of undiagnosed HIV positive women and diagnosed HIV positive women lost to follow-up at each step of the PMTCT program.

The first cascade captures all the steps of the PMTCT program; from the pre-test counseling in antenatal care to the infant prophylaxis (Figure 2). It is based on the data from four studies reporting the uptake of each of the PMTCT interventions [46], [47], [58], [61]. All studies were conducted in low-income sub-Saharan African countries and employed HIV rapid test and single dose nevirapine as ARV prophylaxis for both women and infants. Although most of the women attending antenatal care received pre-test counseling, only 70% of them accepted HIV testing and only 65% of those women received their test result.

About 14% of women attending the antenatal care received an HIV positive result. We estimated that an additional 4% of antenatal care attendees were HIV positive and unaware of their HIV status because they either were not counseled, were not tested, or did not receive test results. This translates to 22% of total HIV positive women attending the antenatal care who were not notified about their HIV status.

Only half of all HIV positive women attending the antenatal care were provided with ARV prophylaxis in antenatal care. Seventeen per cent of all HIV positive women attending the antenatal care were observed or self-reported to have taken the provided ARV prophylaxis at delivery. Similarly, only 17% of the infants born to all HIV positive women that attended antenatal care received ARV prophylaxis.

**Figure 3:**
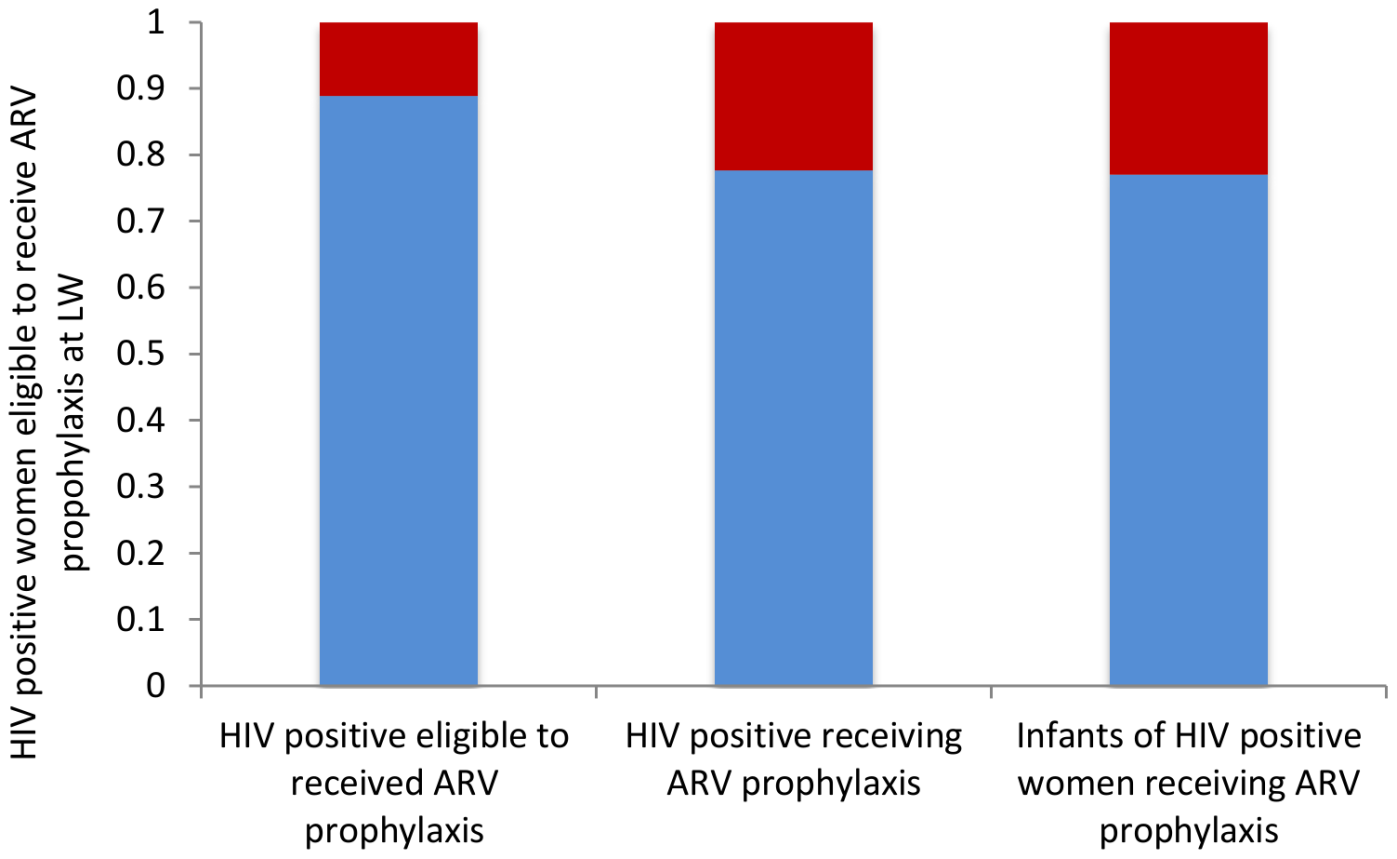
Cascade of integrated PMTCT program at labor ward. Blue bars indicate the proportion of women participating in PMTCT program at labor ward. Red bars show the estimated proportion of eligible HIV positive women at labor ward, not tested (column 1), not receiving ARV prophylaxis (column 2) and proportion of infants born to HIV positive women not receiving ARV prophylaxis (column 3).

The second cascade presents the drop-outs of integrated PMTCT with labor ward (Figure 3) based on the results of three studies providing outcomes for PMTCT interventions provided in labor ward [52], [75], [78]. Ninety eight per cent of eligible women were counseled in labor ward and 89% of them were tested for HIV. Eighty nine per cent of all HIV positive women attending labor ward were diagnosed as infected. We estimated that an additional 11% of all HIV positive were women attending labor ward were not identified as HIV-positive. Seventy eight per cent of all HIV positive women attending labor ward received ARV prophylaxis as well as 78% of the infants. The prevalence of HIV among the HIV tested women in the included studies was 2,8%.

#### Secondary outcomes

Percentage of HIV positive infants born to women living with HIV. Thirteen studies reported the uptake of infant HIV testing. A median of 11% (range 3–18%) of infants were found to be HIV positive [41], [47], [51], [56], [57], [64], [66], [68], [69], [71], [75], [78], [79].

#### Cost analysis

Three studies provided data on the costs of interventions [48], [58], [60].

A 12-month program in Zambia was estimated to cost US$ 221 170. This included start-up costs and recurring costs such as staff salaries and HIV test kits, but excluded expatriate salaries and travel expenses. Subtracting the start-up costs from the overall expenditure, the costs were US$ 9.34 per woman counseled, US$ 12.96 per woman tested and US$ 55.12 for identifying one seropositive woman. This program was funded by the Elizabeth Glaser Pediatric AIDS Foundation [48].

In a South African program the estimated costs per woman enrolled in a postpartum VCT program were US$ 23; these costs included testing women and administering nevirapine to infants whose women were positive [60].

The costs of preventing one HIV child infection was estimated in two studies to range from US$ 848 in a Zambian study (where the initial costs of salaries, training, medical materials, equipment were excluded) [48], to US$ 4444 in a Malawian study (where all the initial costs were included) [58].

#### Impact of integration on human resources

A number of studies reported that the integration of programs led to increased staff workload [51], [52], [59], [74] and that additional personnel was employed [44], [46], [47], [51], [52], [57], [58], [60], [78]. New personnel hired were mostly counselors [44], [46], [58], [60], [78]. In some programs traditional birth attendants, lay counselors or peer-counselors were additionally employed [46], [51], [60]–[65].

One study reported that nurses received compensation because of extra work [63] and in a Zambian study the nurses were allowed to schedule counseling on their off-days for extra pay [48]. Heavy workload on personnel without extra pay was identified as potentially having a negative impact on the success of the program in the long-term [49].

#### Inequalities in access

Three studies identified lack of means as a key barrier to participation in PMTCT program [54], [59], [76]. In a study from Vietnam, there was a low uptake of HIV testing due to service charges. In the same study women were counseled to use infant formula which was provided for free only during the hospital stay but was unaffordable after discharge for most women [76]. In two studies, women opted for a home delivery since they were unable to pay for the maternity services [54], [59].

In some studies women were lost-to follow-up due to lack of transportation [54], [58], [67]. In a Ukrainian study authors identified intravenous drug users as a hard to reach group not attending antenatal care but reporting to hospital in advanced labor [81].

#### Confidentiality

Some studies reported that the confidentiality of counseling was not always achieved due to partially opened doors, interruptions during counseling and lack of space for counseling [50], [59], [60]. Several studies presented activities undertaken to ensure confidentiality of counseling, e.g. additional space was either built in or designated for counseling [46], [47], [58], [62], [63], and patient confidentiality was emphasized during staff training [47], [62], [64], [65], [78].

#### Barriers to implementation, access and sustainability

Several studies identified lack of human, material, and financial resources (with stock-outs of HIV test kits, ARV prophylaxis and ART) as a key barrier to implementation of integrated care in low- and middle-income countries [50], [61]–[64], [67], [74], [76].

Social norms and stigma have been described in many studies as a cause of lower uptake of PMTCT interventions, HIV testing refusal [48], [59], [61], [62], [67], and for preference of breastfeeding even when formula was provided for free [44], [74]. In one study families left their communities after women were identified as HIV positive [64]. In a Nigerian program, HIV positive women who were provided elective caesarean section preferred a vaginal delivery because of cultural aversion to caesarean section [45]. In a study in rural Cameroon, the health worker counseled women to breastfeed their infants instead of using the available formula milk because replacement feeding was considered culturally unacceptable [65].

Complexity of monitoring tools deterred health workers from completing them or following up women [47], [55], [74], [78]. One study reported that health workers were reluctant to give ARV prophylaxis to women in antenatal care as they believed women would not take the medication provided [50].

Late admission to the labor ward sometimes prevented staff from testing women for HIV, receiving the result before delivery and providing them with ARV prophylaxis [70], [75], [84].

Two studies reported that some HIV positive women abandoned their children at hospitals [73], [76]. These infants did not receive ARV prophylaxis or HIV testing, as there were no follow-up services available [76].

The most commonly reported barrier to sustainability was the lack of financial support. Funding provided by the government or/and external donors has been reported as crucial for the success and sustainability of programs [61], [65], [74], [78]. Authors reporting on the PMTCT program from rural Malawi argued that it should be decentralized in the future to improve access. However, they also felt that in that case the provision of free formula would no longer be affordable [58].

Impact of integration on the use of healthcare services integrated with PMTCT interventions was reported in only one study where it did not influence the coverage or flow of women attending antenatal care given as counseling was provided by counselors in separate rooms [46].

The remaining secondary outcomes are presented in Text S8. We presented community involvement strategies employed in the included studies. We also reported data on provision of ART and co-trimoxazole to HIV positive mothers and HIV positive infants after delivery.

## Discussion

This is the first systematic review on the uptake of integrated PMTCT programs in low- and middle-income countries. The existing evidence provides data on PMTCT programs implemented between 1997 and 2006. The overall uptake of PMTCT programs was low. The MTCT rate of HIV in included studies was 11% (range 3–18%). It highlighted many challenges of following through all the steps of the perinatal PMTCT cascade.

Although the uptake of counseling and testing in antenatal care was high, presumably many HIV positive women did not receive ARV prophylaxis in antenatal care and did not deliver at a labor ward. The PMTCT cascade based on the four studies from low-income sub-Saharan African countries with data on the entire perinatal PMTCT program showed a marked loss to follow-up between steps of the program. Only half of HIV positive women received ARV prophylaxis in antenatal care and 17% were known to have taken ARV prophylaxis at delivery (either self-reported or observed at labor ward). We estimated that 22% of all HIV positive women attending antenatal care and 11% of all HIV positive women attending labor ward were not tested or notified about their HIV status. Although the uptake of PMTCT interventions delivered at labor ward was high, it did not reach the 80% target set by UNGASS for HIV testing [16].

In a study performed in four African countries cord blood specimen analysis was used to assess uptake of ARV prophylaxis instead of process indicators. The unadjusted maternal nevirapine coverage was 58% (95% CI, 56–59%) [85]. These findings are strikingly consistent with the findings of this review where 55% (range 22–99%) of HIV positive women received ARV prophylaxis in antenatal care and 52% (range 14–85%) of HIV positive women were known to have taken ARV prophylaxis. However, more recent studies generally report lower ingestion of ARV prophylaxis among HIV positive women within antenatal care which could be due to the more complicated regimens of ARV prophylaxis used lately.

The uptake of ARV prophylaxis at labor ward in women entering PMTCT program at labor ward was 92% compared to 74% of those entering in antenatal care. We assume that this difference is due to late admission of women to the labor ward. Identified HIV positive women arriving at the labor wards were sometimes not provided with ARV prophylaxis because of imminent delivery and lack of time. Further, in the studies reviewed women of unknown HIV status arriving to the labor ward in advanced labor were not tested and diagnosed. Consequently, they were not considered eligible for ARV prophylaxis and are not included in the denominator (HIV positive women delivering at labor ward) misleadingly resulting in higher proportion of women receiving ARV prophylaxis. Furthermore, the higher proportion of participants receiving ARV prophylaxis at labor ward compared to the antenatal care could also be explained by the complexity of the provided ARV prophylaxis. At labor ward, the ARV prophylaxis consisted mostly of single dose nevirapine while in antenatal care women also received more complex regimens. We also observed an excellent correlation of women and infants receiving ARVs at institutional delivery.

This review also showed that not all interventions which should intuitively lead to better outcomes necessarily do so. The opt-out approach increased the proportion of women tested. However, it is unclear if it improves uptake of other PMTCT interventions such as counseling or ARV prophylaxis. Increased awareness of HIV positive serostatus might not lead to more women participating in PMTCT program due to other barriers such as health system failures or fear of stigma. Several studies confirmed higher uptake of HIV testing when opt-out approach was implemented [86]–[89]. Studies from Botswana and Ethiopia reported that opt-out testing lead to better uptake of only testing [86], [88]. A Zimbabwean study reported also an increase in uptake of ARV prophylaxis [89]. Similarly, the implementation of rapid test increased the proportion of women receiving their test result but the difference was not significant when only HIV positive women were considered. Several studies on the effect of rapid test on the PMTCT intervention uptake reported contrasting findings. Rapid test contributed to an increase in the proportion of women who received post-test counseling in two studies [89], [90]. In a Kenyan study significantly fewer HIV positive women were tested with rapid test and accepted referral for other PMTCT interventions compared to those receiving conventional tests [91].

Next, the use of virological test for infants, which means a shorter length of follow up, did not result in a higher proportion of infants tested.

In many countries significant proportion of women delivers at home. One strategy for reaching these women and their infants is the involvement of traditional birth attendants (TBAs). This approach seems to be effective according to two studies from Cameroon [64], [65]. A further Zimbabwean study examining the views of TBAs regarding PMTCT programs, reported that TBAs were willing to participate in most of the PMTCT interventions [92]. Furthermore, TBAs could potentially act as a link to long-term HIV care and treatment services for HIV positive women and children in need of lifelong HAART. This systematic review has a number of strengths. We undertook a comprehensive and extensive search of multiple electronic databases of published and grey literature to identify relevant peer–reviewed studies since the introduction of the PMTCT. We nether used any methodological filters, nor specific search terms for integrated care. We followed methodology principles established by the Cochrane Collaboration and did not apply language restrictions to the search strategy. A detailed and comprehensive data extraction of the selected studies has provided us with a broad range of information and deep insight into the issues surrounding the implementation of integrated PMTCT programs.

Despite our thorough search strategy, we only found studies presenting programs implemented before 2007. It could be argued that the data from our review does not reflect the current, potentially better PMTCT programs which have, in the last few years, received substantial financial investments and could have improved. However, the aforementioned study on uptake in four African countries during 2007 and 2008 showed very similar uptake of PMTCT interventions to the one in our review. In addition, a study presenting national data on PMTCT services in Ethiopia from 2006 to 2010, showed that uptake was low. Although there was significant improvement in HIV counseling and testing uptake during this period, only 53% of known HIV-positive mothers and 38% of known HIV-exposed infants attending PMTCT programs have received ARV prophylaxis in 2009.[88] Furthermore, recent WHO PMTCT guidelines recommend more effective and more complicated regimens. Given the low uptake of simpler PMTCT regimens in the included studies, it is very important to assess the uptake of more recent PMTCT programs following new guidelines. It is of concern that we could not find studies with this information. The potential reason for lack of this information could be that the studies that we found were mostly published with the intention to present the feasibility of the first PMTCT programs. In addition, studies on uptake of PMTCT interventions are mostly based on routine program data and as such could potentially be considered as non-publishable in peer-reviewed journals, especially now that the feasibility of PMTCT programs has been established. However, the low uptake of these programs warrants more recent data which would show the current status of PMTCT services.

The included studies were highly heterogeneous in terms of time of implementation, content and context of implementation (i.e. HIV prevalence, type of HIV epidemic, dominant mode of HIV transmission or countries’ income, training provided to the staff). However, we have used appropriate statistical methods that allowed pooling of the studies. As sample size was limited in each study, we summarized results based on reported frequencies. This yielded higher sample size and higher external validity on cost of biases introduced by the heterogeneity of the studies and their different sample sizes.

We also found the policy context to be quite variable across the included studies. The timing of ARV prophylaxis administration in antenatal care was different and might have also had an impact on the uptake.

Included studies had several limitations which should be considered when interpreting these findings. The characteristics of participants were poorly reported in most of the studies. Additionally, there was a paucity of information about the setting of integrated PMTCT care, the nature of intervention and the content of the training provided to the staff. Although this systematic review was focused on the integration of service delivery, we noticed the general lack of data on integration of other aspects of the health system (e.g. health information systems, leadership, financing etc.). The studies were mostly based on single health facilities and given high levels of short-term migration of women at the time of delivery, might have failed to capture the potential uptake of ARV prophylaxis at other facilities. Although we aimed to capture the proportion of women receiving the full ARV prophylaxis regimen in the antenatal care, in two included studies we only found data on the proportion of women who started it. Most of the studies reported who funded the PMTCT programs, but there was no information on the proportion of financing from domestic versus international sources, per person or total annual financing for PMTCT as a proportion of the HIV or health budget. Many studies did not provide clear, quantitative data on the proportion of women counseled on infant feeding, costs, impact on maternal and child healthcare services attendance and stigma. The information on follow-up of HIV positive women and infants after the delivery was scarce. We also wanted to present other aspects of quality of care in the integrated programs but only found information on confidentiality of counseling. However, these studies were primarily focused on perinatal programs, presented programs implemented before the new WHO guidelines on follow-up. Our ability to detect statistically significant difference in subgroup analyses was limited by the low number of studies reporting on coverage of individual interventions.

The findings of our review provide most timely information on weaknesses in and the uptake of PMTCT cascade at the time when PMTCT is receiving a reinvigorated commitment and scaling-up is the highest priority of all main players in global health from governments to global agencies and financing institutions. The findings are important and inform the global estimates of the impact of PMTCT interventions as we provide the most up to date status of how initial coverage levels translate to effective prophylaxis for prevention of MTCT of HIV. Models used to estimate coverage and impact of PMTCT interventions need to factor adequately for the 'substantial' attrition rates to produce a more realistic estimate of coverage impact.

If the ambition of HIV-free and AIDS-free generation is to be achieved by 2015 [93], implementation strategies for PMTCT programs need to receive much greater attention for each step of the cascade.

Future research should in particular focus on the strategies to improve access to maternal and child healthcare services (perhaps through more systematic use of mobile phones) and to provide PMTCT interventions to HIV positive women delivering at home. Given that the main challenge is reaching the women who are not attending antenatal care or delivering at a labor ward, it is necessary to evaluate outreach options and the use of healthcare services other than maternal and child healthcare programs as entry points to the PMTCT programs.

## Supporting Information

Figure S1 **Year of initiation of the described PMTCT programs.**
(DOCX)

Table S1 **Framework for the integration of PMTCT program with maternal and child healthcare Services.**
(DOCX)

Table S2 **Data extraction headings.**
(DOCX)

Table S3 **Search results by source.**
(DOCX)

Table S4 **Excluded studies and reasons of exclusion.**
(DOCX)

Table S5 **HIV prevalence in the included studies.**
(DOCX)

Table S6 **Number of studies providing various types of ARV prophylaxis.**
(DOCX)

Text S1 **The World Health Organization definition of PMTCT program.**
(DOCX)

Text S2 **List of primary and secondary outcomes.**
(DOCX)

Text S3 **Exclusion criteria.**
(DOCX)

Text S4 **Search strategy.**
(DOCX)

Text S5 **Description of denominators.**
(DOCX)

Text S6 **Description of setting, participants and staff from the included studies.**
(DOCX)

Text S7 **Description of PMTCT interventions.**
(DOCX)

Text S8 **Data about subgroup analysis, community involvement and maternal and infant follow-up.**
(DOCX)
