# Supplementary material for: The Uptake of Integrated Perinatal Prevention of Mother-to-Child HIV Transmission Programs in Low- and Middle-Income Countries: A Systematic Review
Source: PLoS One. 2013 Mar 6;8(3):e56550. doi: 10.1371/journal.pone.0056550 (PMC3590218; doi:10.1371/journal.pone.0056550)
Supplement: Figure S1 — Year of initiation of the described PMTCT programs. (DOCX) [file pone.0056550.s001.docx]

**Figure S1: Year of initiation of the described PMTCT programs**
