# Supplementary material for: The Uptake of Integrated Perinatal Prevention of Mother-to-Child HIV Transmission Programs in Low- and Middle-Income Countries: A Systematic Review
Source: PLoS One. 2013 Mar 6;8(3):e56550. doi: 10.1371/journal.pone.0056550 (PMC3590218; doi:10.1371/journal.pone.0056550)
Supplement: Table S1 — Framework for the integration of PMTCT program with maternal and child healthcare Services. (DOCX) [file pone.0056550.s002.docx]

Table S1: Framework for the integration of PMTCT program with maternal and child healthcare services

| **PMTCT interventions** | **Healthcare services** | | |
| --- | --- | --- | --- |
|  | **ANC** | **LW** | **PNC/Infant FU** |
| Testing women for HIV | + | + |  |
| ART (started as part of PMTCT) | + |  |  |
| Women ARV prophylaxis | + | + |  |
| Safe delivery | +^a^ | + |  |
| Infant ARV prophylaxis | +^b^ | + |  |
| Infant feeding counseling | + | + | + |
| Testing infants for HIV | +^c^ | +^c^ | + |

Abbreviations: ANC, antenatal care; FU, follow-up; LW, labor ward; PMTCT, prevention of mother to child transmission; PNC=postnatal care.

^a^Women referred to a hospital providing safe delivery.

^b^Women provided with antiretroviral prophylaxis for the infant.

^c^Women told to return to have the infants tested.
