# Supplementary material for: The Uptake of Integrated Perinatal Prevention of Mother-to-Child HIV Transmission Programs in Low- and Middle-Income Countries: A Systematic Review
Source: PLoS One. 2013 Mar 6;8(3):e56550. doi: 10.1371/journal.pone.0056550 (PMC3590218; doi:10.1371/journal.pone.0056550)
Supplement: Table S2 — Data extraction headings. (DOCX) [file pone.0056550.s003.docx]

**Table S2: Data extraction headings**

- Author
- Publication year
- Language
- Country
- World Bank Income group
- Type of publication
- Study design
- Study duration
- Number of integrated facilities
- Site of Integration (ANC, LW, PNC, other)
- Mode of delivery of the intervention (Single point, referral)
- Description of different program parameters (personnel , service fees, training, other)
- Characteristics of enrolled participants
- Primary outcome measures
- Secondary outcome measures
- Type of HIV tests
- Methods of feeding counseling
- Modes of safe delivery
- Type of ARV prophylaxis and therapy
- Adverse outcomes of integration
- Barriers to Integration and sustainability
- Comments
