# Supplementary material for: The Uptake of Integrated Perinatal Prevention of Mother-to-Child HIV Transmission Programs in Low- and Middle-Income Countries: A Systematic Review
Source: PLoS One. 2013 Mar 6;8(3):e56550. doi: 10.1371/journal.pone.0056550 (PMC3590218; doi:10.1371/journal.pone.0056550)
Supplement: Table S3 — Search results by source. (DOCX) [file pone.0056550.s004.docx]

**Table S3: Search results by source**

| **Database** | **Results** |
| --- | --- |
| AIDS Education Global Information System (AEGIS) | 1000 |
| British Library Catalogue (BETA) | 415 |
| CLINICALTRIALS.gov | 3362 |
| [Cochrane Central Register of Controlled Trials (CENTRAL)](http://www.mrw.interscience.wiley.com/cochrane/cochrane_clcentral_articles_fs.html) | 260 |
| Cochrane Database of Systematic Reviews | 78 |
| Cumulative Index to Nursing and Allied Health Literature (CINAHL) | 651 |
| Database of Abstracts of Reviews on Effects | 11 |
| Education Resources Information Centre (ERIC) | 16 |
| EMBASE | 4910 |
| Global Health (CAB Abstracts) | 1421 |
| Google Scholar | 500 |
| MEDLINE | 5322 |
| New York Academy of Medicine Grey Literature Collection | 177 |
| OpenSIGLE | 144 |
| Population Information Online (POPLINE) | 4971 |
| ProQuest Dissertations and Theses Database | 449 |
| PsychINFO | 525 |
| Sociological Abstracts | 173 |
| U.S. National Library of Medicine’s (NLM) Gateway system | 2680 |
| WHO International Clinical Trials Registry Platform (WHO ICTRP) | 59 |
| World Health Organization’s The Global Health Library | 1530 |
| **Total** | 28 654 |
