# Supplementary material for: The Uptake of Integrated Perinatal Prevention of Mother-to-Child HIV Transmission Programs in Low- and Middle-Income Countries: A Systematic Review
Source: PLoS One. 2013 Mar 6;8(3):e56550. doi: 10.1371/journal.pone.0056550 (PMC3590218; doi:10.1371/journal.pone.0056550)
Supplement: Table S4 — Excluded studies and reasons of exclusion. (DOCX) [file pone.0056550.s005.docx]

**Table S4: Excluded studies and reasons for exclusion**

| **Author, Year** | **Reason for exclusion** |
| --- | --- |
| Amornwichet et al, 2002[[1](#_ENREF_1)] | Description of non-integrated care or integration is unclear |
| Arreskov et al, 2010[[2](#_ENREF_2)] |  |
| Coetzee et al, 2005[[3](#_ENREF_3)] |  |
| Dandona, L. et al, 2008[[4](#_ENREF_4)] |  |
| Ginsburg et al, 2007[[5](#_ENREF_5)] |  |
| Kakabadze et al, 2008[[6](#_ENREF_6)] |  |
| Kanshana S and Simonds RJ, 2002[[7](#_ENREF_7)] |  |
| Marazzi et al, 2007[[8](#_ENREF_8)] |  |
| Matida et al, 2005[[9](#_ENREF_9)] |  |
| Mazia et al, 2009[[10](#_ENREF_10)] |  |
| Peltzer K et al, 2008[[11](#_ENREF_11)] |  |
| Quian J et al, 2005[[12](#_ENREF_12)] |  |
| Sherman et al, 2004[[13](#_ENREF_13)] |  |
| Simpore et al, 2006[[14](#_ENREF_14)] |  |
| Thorne et al, 2009 [[15](#_ENREF_15)] |  |
| Adegoke  Cavalcante et al, 2008[[16](#_ENREF_16)] | Assessing knowledge and/or factors associated with the uptake of PMTCT services |
| Etiebet et al, 2004[[17](#_ENREF_17)] |  |
| Fabbro et al, 2002[[18](#_ENREF_18)] |  |
| Goldani et al, 2003[[19](#_ENREF_19)] |  |
| Holmes et al, 2008[[20](#_ENREF_20)] |  |
| Karamagi et al, 2006[[21](#_ENREF_21)] |  |
| Kizito et al, 2008[[22](#_ENREF_22)] |  |
| Kongnyuy et al, 2009[[23](#_ENREF_23)] |  |
| Lemos et al, 2005 [[24](#_ENREF_24)]  Mathur et al, 2008[[25](#_ENREF_25)] |  |
| Mbonye et al, 2009[[26](#_ENREF_26)] |  |
| Mbonye et al, 2010[[27](#_ENREF_27)] |  |
| Megazzini et al, 2009[[28](#_ENREF_28)] |  |
| Mkwanazi et al, 2008[[29](#_ENREF_29)] |  |
| Piwoz et al, 2005[[30](#_ENREF_30)] |  |
| Rollins et al, 2009[[31](#_ENREF_31)] |  |
| Turchi et al, 2007[[32](#_ENREF_32)] |  |
| Amaral et al, 2007[[33](#_ENREF_33)] | Reporting only on outcomes we defined as secondary, clinical outcomes or outcomes out of the scope of this review |
| Chee, G and K. Smith, 2006[[34](#_ENREF_34)]  Chege, J.N. and I. Askew, 2005[[35](#_ENREF_35)]  Cotton et al, 2006[[36](#_ENREF_36)] |  |
| Duran et al, 2006[[37](#_ENREF_37)] |  |
| Essomo et al, 2008[[38](#_ENREF_38)] |  |
| Ferguson et al, 2009[[39](#_ENREF_39)] |  |
| Gumbo et al, 2010 [[40](#_ENREF_40)] |  |
| Gupta et al, 2007 [[41](#_ENREF_41)] |  |
| Homsy et al, 2010 [[42](#_ENREF_42)] |  |
| Hoog et al, 2005[[43](#_ENREF_43)]  Hussein, I. and A. Ahmed, 2009 [[44](#_ENREF_44)] |  |
| Hylton-Kong et al, 2008 [[45](#_ENREF_45)]  Kasenga et al, 2009[[46](#_ENREF_46)]  Killam et al, 2010[[47](#_ENREF_47)]  Megazzini et al, 2010 [[48](#_ENREF_48)] |  |
| Moses et al, 2008 [[49](#_ENREF_49)] |  |
| Munguti et al,2006[[50](#_ENREF_50)]  Njom et al, 2007 [[51](#_ENREF_51)] |  |
| Price et al, 2009 [[52](#_ENREF_52)] |  |
| Sagay et al, 2006 [[53](#_ENREF_53)] |  |
| Simba et al, 2010 [[54](#_ENREF_54)]  Stinson et al, 2010 [[55](#_ENREF_55)] |  |
| Van Der Merwe et al, 2006 [[56](#_ENREF_56)] |  |
| Adegoke et al, 2009[[57](#_ENREF_57)]  Baek, C and N. Rutenberg et al, 2010[[58](#_ENREF_58)]  Berer, 1999[[59](#_ENREF_59)] | Quantitative analysis was not possible |
| Chen et al, 2010[[60](#_ENREF_60)] |  |
| Colvin et al, 2007[[61](#_ENREF_61)] |  |
| Dube et al, 2008[[62](#_ENREF_62)] |  |
| Horwood et al, 2010 [[63](#_ENREF_63)] |  |
| Jackson et al, 2007 [[64](#_ENREF_64)] |  |
| Lim et al, 2010[[65](#_ENREF_65)] |  |
| Luo et al, 2007[[66](#_ENREF_66)] |  |
| Micek, 2005[[67](#_ENREF_67)] |  |
| Millogo Traore et al, 2008[[68](#_ENREF_68)] |  |
| Nakakeeto, O. N. and L. Kumaranayake, 2009 [[69](#_ENREF_69)] |  |
| Orie et al, 2009[[70](#_ENREF_70)] |  |
| Smith et al, 2004[[71](#_ENREF_71)] |  |
| Stringer et al, 2005[[72](#_ENREF_72)] |  |
| Teeraratkul et al, 2005[[73](#_ENREF_73)] |  |
| Thaineua et al, 2001[[74](#_ENREF_74)] |  |
| Urban, M. and M. Chersich, 2004[[75](#_ENREF_75)] |  |
| Butsashvili et al, 2008[[76](#_ENREF_76)] | Un-accessed Studies |
| Christie, C. D., 2004[[77](#_ENREF_77)] |  |
| Fabiani et al, 2007[[78](#_ENREF_78)] |  |
| Johnson et al, 2004[[79](#_ENREF_79)] |  |
| Lawson-Evi et al, 2010 [[80](#_ENREF_80)] |  |
| Trujillo, L. A., 2005[[81](#_ENREF_81)] |  |
| Ugochukwu, E. F. and S. O. Kanu, 2010[[82](#_ENREF_82)] |  |
| Vu, T. C., 2010[[83](#_ENREF_83)] |  |
| Wang et al, 2009[[84](#_ENREF_84)] |  |
| Kirere et al, 2007[[85](#_ENREF_85)]  Perez et al, 2004[[86](#_ENREF_86)] | Secondary reports |

1. Amornwichet P, Teeraratkul A, Simonds RJ, Naiwatanakul T, Chantharojwong N, et al. (2002) Preventing mother-to-child HIV transmission: the first year of Thailand's national program. JAMA 288: 245-248.

2. Arreskov A, Minja E, Theilgaard Z, Mandara C, Gerstoft J, et al. (2010) Referral success among HIV-infected women and HIV-exposed children referred for monitoring and treatment in Tanga, Tanzania. Int Health 2: 36-41.

3. Coetzee D, Hilderbrand K, Boulle A, Draper B, Abdullah F, et al. (2005) Effectiveness of the first district-wide programme for the prevention of mother-to-child transmission of HIV in South Africa. Bull World Health Organ 83: 489-494.

4. Dandona L, Kumar SG, Ramesh YK, Rao MC, Marseille E, et al. (2008) Outputs, cost and efficiency of public sector centres for prevention of mother to child transmission of HIV in Andhra Pradesh, India. BMC Health Serv Res 8: 26.

5. Ginsburg AS, Hoblitzelle CW, Sripipatana TL, Wilfert CM (2007) Provision of care following prevention of mother-to-child HIV transmission services in resource-limited settings. AIDS 21: 2529-2532.

6. Kakabadze T, Asatiani T, Bokhua Z, Shermadini K, Lanchava N (2008) Implementation of PMTCT in Georgia. Georgian Med News: 23-28.

7. Kanshana S, Simonds RJ (2002) National program for preventing mother-child HIV transmission in Thailand: successful implementation and lessons learned. AIDS 16: 953-959.

8. Marazzi CM, Germano P, Liotta G, Guidotti G, Loureiro S, et al. (2007) Implementing anti-retroviral triple therapy to prevent HIV mother-to-child transmission: a public health approach in resource-limited settings. Eur J Pediatr 166: 1305-1307.

9. Matida LH, Da Silva MH, Tayra A, Succi RC, Gianna MC, et al. (2005) Prevention of mother-to-child transmission of HIV in Sao Paulo State, Brazil: an update. AIDS 19 Suppl 4: S37-S41.

10. Mazia G, Narayanan I, Warren C, Mahdi M, Chibuye P, et al. (2009) Integrating quality postnatal care into PMTCT in Swaziland. GlobPublic Health 4: 253-270.

11. Peltzer K, Mosala T, Dana P, Fomundam H (2008) Follow-up survey of women who have undergone a prevention of mother-to-child transmission program in a resource-poor setting in South Africa. J Assoc Nurses AIDS Care 19: 450-460.

12. Quian J, Visconti A, Gutierrez S, Galli A, Maturo M, et al. (2005) [Detection of HIV infection in pregnant women by rapid testing: a successful strategy to reduce its vertical transmission]. Rev Chilena Infectol 22: 321-326.

13. Sherman GG, Jones SA, Coovadia AH, Urban MF, Bolton KD (2004) PMTCT from research to reality--results from a routine service. S Afr Med J 94: 289-292.

14. Simpore J, Pietra V, Savadogo A, Pignatelli S, Nikiema JB, et al. (2006) Reduction of mother-to-child transmission of HIV at Saint Camille Medical Centre in Burkina Faso. J Med Virol 78: 148-152.

15. Thorne C, Semenenko I, Pilipenko T, Malyuta R (2009) Progress in prevention of mother-to-child transmission of HIV infection in Ukraine: results from a birth cohort study. BMC Infect Dis 9: 40.

16. Cavalcante MdS, Silveira ACBd, Ribeiro AMdS, Ramos Junior AN (2008) Prevention of vertical transmission of HIV: analysis of the adhesion to prophylactic measures in a reference maternity in the city of Fortaleza, State of Ceara, Brasil. Revista Brasileira de Saude Materno Infantil 8: 473-479.

17. Etiebet MA, Fransman D, Forsyth B, Coetzee N, Hussey G (2004) Integrating prevention of mother-to-child HIV transmission into antenatal care: learning from the experiences of women in South Africa. AIDS Care 16: 37-46.

18. Fabbro MM, Reis MB, Freitas GM, Lindenberg AS, Dobbro RR, et al. (2002) Seroprevalence of the indigenous pregnant woman HIV-infected at Mato Grosso do Sul, Brazil. Int Conf AIDS 14: abstract.

19. Goldani MZ, Giugliani ER, Scanlon T, Rosa H, Castilhos K, et al. (2003) Voluntary HIV counseling and testing during prenatal care in Brazil. Rev Saude Publica 37: 552-558.

20. Holmes C, Preko P, Bolds R, Baidoo J, Jolly P (2008) Acceptance of Voluntary Counselling, Testing and Treatment for HIV Among Pregnant Women in Kumasi, Ghana. Ghana Med J 42: 8-15.

21. Karamagi CA, Tumwine JK, Tylleskar T, Heggenhougen K (2006) Antenatal HIV testing in rural eastern Uganda in 2003: incomplete rollout of the prevention of mother-to-child transmission of HIV programme? BMC Int Health Hum Rights 6: 6.

22. Kizito D, Woodburn PW, Kesande B, Ameke C, Nabulime J, et al. (2008) Uptake of HIV and syphilis testing of pregnant women and their male partners in a programme for prevention of mother-to-child HIV transmission in Uganda. Trop Med Int Health 13: 680-682.

23. Kongnyuy EJ, Mbu ER, Mbopi-Keou FX, Fomulu N, Nana PN, et al. (2009) Acceptability of intrapartum HIV counselling and testing in Cameroon. BMC Pregnancy Childbirth 9: 9.

24. Lemos LMDd, Gurgel RQ, Fabbro ALd (2005) Prevalence of infection with in unified health care system maternities. Revista Brasileira de Ginecologia e Obstetricia 27: 32-36.

25. Mathur M, Taklikar S, Turbadkar D (2008) Study of HIV seroprevalence and sociodemographic patterns in antenatal cases and vertical transmission to neonates letter. Indian J Med Microbiol 26: 289-280.

26. Mbonye AK, Hansen KS, Wamono F, Magnussen P (2009) Increasing access to prevention of mother-to-child transmission of HIV services through the private sector in Uganda. Sex Transm Infect 85: 534-539.

27. Mbonye AK, Hansen KS, Wamono F, Magnussen P (2010) Integration of malaria and HIV/AIDS prevention services through the private sector in Uganda. Int Health 2: 52-58.

28. Megazzini KM, Chintu N, Vermund SH, Redden DT, Krebs DW, et al. (2009) Predictors of rapid HIV testing acceptance and successful nevirapine administration in Zambian labor wards. J Acquir Immune Defic Syndr 52: 273-279.

29. Mkwanazi NB, Patel D, Newell ML, Rollins NC, Coutsoudis A, et al. (2008) Rapid testing may not improve uptake of HIV testing and same day results in a rural South African community: a cohort study of 12,000 women. PLoS One 3: e3501.

30. Piwoz EG, Iliff PJ, Tavengwa N, Gavin L, Marinda E, et al. (2005) An education and counseling program for preventing breast-feeding-associated HIV transmission in Zimbabwe: design and impact on maternal knowledge and behavior. J Nutr 135: 950-955.

31. Rollins N, Mzolo S, Moodley T, Esterhuizen T, van RH (2009) Universal HIV testing of infants at immunization clinics: an acceptable and feasible approach for early infant diagnosis in high HIV prevalence settings. AIDS 23: 1851-1857.

32. Turchi MD, Duarte LS, Martelli CM (2007) Mother-to-child transmission of HIV: risk factors and missed opportunities for prevention among pregnant women attending health services in Goiania, Goias State, Brazil. Cad Saude Publica 23 Suppl 3: S390-S401.

33. Amaral E, Assis-Gomes F, Milanez H, Cecatti JG, Vilela MM, et al. (2007) [Timely implementation of interventions to reduce vertical HIV transmission: a successful experience in Brazil]. Rev Panam Salud Publica 21: 357-364.

34. Chee G, Smith K (2006) Cost and effectiveness analysis of the Ndola Demonstration Project in Zambia: Bethesda, Maryland, Abt Associates, 2006 Feb. [47] p.

35. Chege JN, Beksinska M (2004) The effectiveness of an intervention to integrate HIV and STI prevention information into antenatal care services in rural clinics in South Africa. Int ConfAIDS 15: abstract.

36. Cotton M, Rabie H, Weber H, Lottering G, Nachman S (2006) A window into health care of HIV exposed infants in a community with high prevalence of HIV and tuberculosis: lessons from screening for a prospective clinical trial. AIDS 16: abstract.

37. Duran AS, Ivalo SA, Hakim A, Masciottra FM, Zlatkes R, et al. (2006) Prevention of mother to child HIV transmission. Medicina 66: 24-30.

38. Essomo Megnier-Mbo M, Meye JF, Belembaogo E, Engoghan E, Ondo A (2008) Prevention of mother-to-child transmission of HIV in Gabon. The problem of children lost to follow-up. Arch Pediatr 15: 1703-1704.

39. Ferguson YO, Eng E, Bentley M, Sandelowski M, Steckler A, et al. (2009) Evaluating nurses' implementation of an infant-feeding counseling protocol for HIV-infected mothers: The Ban Study in Lilongwe, Malawi. AIDS Educ Prev 21: 141-155.

40. Gumbo FZ, Kurewa NE, Quintoline G, Kandawasvika, Duri K, et al. (2010) Rising mother-to-child HIV transmission in a resource-limited breastfeeding population. Trop Doct 40: 70-73.

41. Gupta A, Gupte N, Sastry J, Bharucha KE, Bhosale R, et al. (2007) Mother-to-child transmission of HIV among women who chose not to exclusively breastfeed their infants in Pune, India. Indian J Med Res 126: 131-134.

42. Homsy J, Moore D, Barasa A, Were W, Likicho C, et al. (2010) Breastfeeding, mother-to-child HIV transmission, and mortality among infants born to HIV-Infected women on highly active antiretroviral therapy in rural Uganda. J Acquir Immune Defic Syndr 53: 28-35.

43. Hoog AHvt, Mbori-Ngacha DA, Marum LH, Otieno JA, Misore AO, et al. (2005) Preventing mother-to-child transmission of HIV in Western Kenya: operational issues. J Acquir Immune Defic Syndr 40: 344-349.

44. Hussein I, Ahmed A (2009) Status of ANC-linked HIV counseling and testing as an intervention for PMTCT in public health facilities in Addis Ababa: quality of HIV counseling given to pregnant women for PMTCT. Ethiopian Journal of Health Development 23: 190-198.

45. Hylton-Kong T, Alveranga H, Norrine D, Harris H, Daubon G (2008) Contact investigation in the prevention of mother-to-child transmission of HIV comparing urban and rural outcomes in Jamaica. West Indian Med J 57: 282-286.

46. Kasenga F, Byass P, Emmelin M, Hurtig AK (2009) The implications of policy changes on the uptake of a PMTCT programme in rural Malawi: first three years of experience. Glob Health Action 2.

47. Killam WP, Tambatamba BC, Chintu N, Rouse D, Stringer E, et al. (2010) Antiretroviral therapy in antenatal care to increase treatment initiation in HIV-infected pregnant women: A stepped-wedge evaluation. AIDS 24: 85-91.

48. Megazzini KM, Sinkala M, Vermund SH, Redden DT, Krebs DW, et al. (2010) A cluster-randomized trial of enhanced labor ward-based PMTCT services to increase nevirapine coverage in Lusaka, Zambia. AIDS 24: 447-455.

49. Moses A, Zimba C, Kamanga E, Nkhoma J, Maida A, et al. (2008) Prevention of mother-to-child transmission: program changes and the effect on uptake of the HIVNET 012 regimen in Malawi. AIDS 22: 83-87.

50. Munguti N, Mokua M, Homan R, Birungi H (2006) Cost analysis of reproductive health services in PCEA Chogoria Hospital, Kenya: Nairobi, Kenya, Population Council, Frontiers in Reproductive Health, 2006 Jun. 11 p.

51. Njom NA, Penda I, Same EC, Tene G, Tsague L (2007) Is exclusive artificial feeding feasible at 6 months post partum in Cameroon urban areas for HIV-exposed infants? J Trop Pediatr 53: 438-439.

52. Price JE, Leslie JA, Welsh M, Binagwaho A (2009) Integrating HIV clinical services into primary health care in Rwanda: a measure of quantitative effects. AIDS Care 21: 608-614.

53. Sagay AS, Musa J, Adewole AS, Imade GE, Ekwempu CC, et al. (2006) Rapid HIV testing and counselling in labour in a northern Nigerian setting. Afr J ReprodHealth 10: 76-80.

54. Simba D, Kamwela J, Mpembeni R, Msamanga G (2010) The impact of scaling-up prevention of mother-to-child transmission (PMTCT) of HIV infection on the human resource requirement: the need to go beyond numbers. Int J Health Plann Manage 25: 17-29.

55. Stinson K, Boulle A, Coetzee D, Abrams EJ, Myer L (2010) Initiation of highly active antiretroviral therapy among pregnant women in Cape Town, South Africa. Trop Med Int Health 15: 825-832.

56. Van Der Merwe K, Chersich MF, Technau K, Umurungi Y, Conradie F, et al. (2006) Integration of antiretroviral treatment within antenatal care in Gauteng Province, South Africa. J Acquir Immune Defic Syndr 43: 577-581.

57. Adegoke OD, Basir Z, Jumare J, Sani S, Enzama R, et al. (2009) Outcome of early infant diagnosis in exposed infants in Northern Nigeria. Journal of Acquired Immune Deficiency Syndromes 51: 172.

58. Baek C, Rutenberg N (2010) Implementing programs for the prevention of mother-to-child HIV transmission in resource-constrained settings: Horizons studies, 1999-2007. Public Health Rep 125: 293-304.

59. Berer M (1999) Reducing perinatal HIV transmission in developing countries through antenatal and delivery care, and breastfeeding: supporting infant survival by supporting women's survival. Bulletin of the World Health Organization 77: 871-877.

60. Chen JY, Ogwu AC, Svab P, Lockman S, Moffat HJ, et al. (2010) Antiretroviral treatment initiation among HIV-infected pregnant women with low CD4(+) cell counts in Gaborone, Botswana. J Acquir Immune Defic Syndr 54: 102-106.

61. Colvin M, Chopra M, Doherty T, Jackson D, Levin J, et al. (2007) Operational effectiveness of single-dose nevirapine in preventing mother-to-child transmission of HIV. Bull World Health Organ 85: 466-473.

62. Dube S, Boily MC, Mugurungi O, Mahomva A, Chikhata F, et al. (2008) Estimating vertically acquired HIV infections and the impact of the prevention of mother-to-child transmission program in Zimbabwe: insights from decision analysis models. J Acquir Immune Defic Syndr 48: 72-81.

63. Horwood C, Haskins L, Vermaak K, Phakathi S, Subbaye R, et al. (2010) Prevention of mother to child transmission of HIV (PMTCT) programme in KwaZulu-Natal, South Africa: an evaluation of PMTCT implementation and integration into routine maternal, child and women's health services. Trop Med Int Health.

64. Jackson DJ, Chopra M, Doherty TM, Colvin MS, Levin JB, et al. (2007) Operational effectiveness and 36 week HIV-free survival in the South African programme to prevent mother-to-child transmission of HIV-1. AIDS 21: 509-516.

65. Lim Y, Kim JY, Rich M, Stulac S, Niyonzima JB, et al. (2010) Improving Prevention of Mother-to-Child Transmission of HIV Care and Related Services in Eastern Rwanda. PLoS Med 7: e1000302.

66. Luo C, Akwara P, Ngongo N, Doughty P, Gass R, et al. (2007) Global progress in PMTCT and paediatric HIV care and treatment in low- and middle-income countries in 2004-2005. Reprod Health Matters 15: 179-189.

67. Micek M (2005) Integrating TB and HIV care in Mozambique: lessons from an HIV clinic in Beira: Seattle, Washington, Health Alliance International, 2005 Sep. 14 p.

68. Millogo-Traore F, Lankoande J, Ouoba R (2008) [Mother to child prevention against HIV in the Health District of Kossodo (Burkina Faso)]. Rev Med Brux 29: 153-158.

69. Nakakeeto ON, Kumaranayake L (2009) The global strategy to eliminate HIV infection in infants and young children: A seven-country assessment of costs and feasibility. AIDS 23: 987-995.

70. Orie EF, Songca PP, Moodley J (2009) An audit of PMTCT services at a regional hospital in South Africa. SA Fam Pract 51: 492-495.

71. Smith J, Posokhova S, Nizova N, Tyapkin G, Schecter K, et al. (2004) The Odessa "Plus-PMTCT-Plus" model: replicable reorganization of a health care delivery system for effective prevention of HIV infection among infants in resource-limited settings in Eurasia. Int Conf AIDS 15: abstract.

72. Stringer JS, Sinkala M, Maclean CC, Levy J, Kankasa C (2005) Effectiveness of a city-wide program to prevent mother-to-child HIV transmission in Lusaka, Zambia. Aids 19: 1309-1315.

73. Teeraratkul A, Simonds RJ, Asavapiriyanont S, Chalermchokcharoenkit A, Vanprapa N, et al. (2005) Evaluating programs to prevent mother-to-child HIV transmission in two large Bangkok hospitals, 1999-2001. J Acquir Immune Defic Syndr 38: 208-212.

74. Thaineua V, Kanshana S, Thewanda D, Amornwichet P, Kullerk N (2001) Evaluation of a regional pilot program to prevent mother-infant HIV transmission -- Thailand, 1998-2000. MMWR Morbidity and Mortality Weekly Report 50: 599-603.

75. Urban M, Chersich M (2004) Acceptability and utilisation of voluntary HIV testing and nevirapine to reduce mother-to-child transmission of HIV-1 integrated into routine clinical care. S Afr Med J 94: 362-366.

76. Butsashvili M, Preble E, Kamkamidze G, Robinson J, Chubinishvili O, et al. (2008) Uptake of an HIV voluntary counseling and testing program for pregnant women in Georgia. AIDS Care 20: 1125-1127.

77. Christie CD (2004) A paediatric and perinatal HIV / AIDS leadership initiative in Kingston, Jamaica. West Indian Medical Journal 53 Spec No: 283-292.

78. Fabiani M, Cawthorne A, Nattabi B, Ayella EO, Ogwang M (2007) Investigating factors associated with uptake of HIV voluntary counseling and testing among pregnant women living in North Uganda. AIDS Care 19: 733-739.

79. Johnson N, Mullings AA, Harvey KM, Alexander G, McDonald D (2004) HIV seroprevalence, uptake of interventions to reduce mother-to-child transmission and birth outcomes in greater Kingston, Jamaica. West Indian Medical Journal 53 Spec No: 297-302.

80. Lawson-Evi K, Mouhari-Toure A, Tchama R, Akakpo SA, Atakouma DY, et al. (2010) [Fate of children born to HIV positive mothers followed in the context of preventing mother-to-child transmission of HIV in Togo. Study of 1042 infants.]. Bull Soc PatholExot.

81. Trujillo LA (2005) HIV testing as part of routine antenatal care in Bangkok, Thailand [3170388]. United States -- Louisiana: Tulane University. 209 p.

82. Ugochukwu EF, Kanu SO (2010) Early Infant Diagnosis of HIV Infection in Southeastern Nigeria: Prevalence of HIV Infection Among HIV-Exposed Babies. West Afr J Med 29: 3-7.

83. Vu TC (2010) A costing exercise of provision of prevention of HIV transmission from mother to child services in Vietnam. Dissertation Abstracts International: Section B: The Sciences and Engineering 70: 4122.

84. Wang LH, Fang LW, Wang Q, Jiang Y, Mo Y, et al. (2009) [The change trend of mother-to-child transmission rate of HIV-1 during 2005-2007 in some areas of China]. Zhonghua Yu Fang Yi Xue Za Zhi 43: 984-987.

85. Kirere MM, Lepage P (2007) Feasibility of HIV prophylaxis with nevirapine in a northeastern rural area of the Democratic Republic of the Congo. Cahiers Sante 17: 97-101.

86. Perez F, Orne-Gliemann J, Mukotekwa T, Miller A, Glenshaw M (2004) Prevention of mother to child transmission of HIV: evaluation of a pilot programme in a district hospital in rural Zimbabwe. BMJ British Medical Journal 329: 1147-1150.
