# Supplementary material for: The Uptake of Integrated Perinatal Prevention of Mother-to-Child HIV Transmission Programs in Low- and Middle-Income Countries: A Systematic Review
Source: PLoS One. 2013 Mar 6;8(3):e56550. doi: 10.1371/journal.pone.0056550 (PMC3590218; doi:10.1371/journal.pone.0056550)
Supplement: Table S5 — HIV prevalence in the included studies. (DOCX) [file pone.0056550.s006.docx]

**Table S5: HIV prevalence in the included studies**

| HIV Prevalence | Africa | Europe | South-East Asia | The Americas | The Western Pacific | Grand Total |
| --- | --- | --- | --- | --- | --- | --- |
| <1% |  | 2 | 4 | 4 |  | 10 |
| 1-5% | 3 | 1 | 1 | 2 | 1 | 8 |
| 5-10% | 12 |  |  |  |  | 12 |
| 10-15% | 2 |  |  |  |  | 2 |
| 15-20% | 2 |  |  |  |  | 2 |
| 20-30% | 6 |  |  |  |  | 6 |
| >30% | 1 |  |  |  |  | 1 |
