# Supplementary material for: The Uptake of Integrated Perinatal Prevention of Mother-to-Child HIV Transmission Programs in Low- and Middle-Income Countries: A Systematic Review
Source: PLoS One. 2013 Mar 6;8(3):e56550. doi: 10.1371/journal.pone.0056550 (PMC3590218; doi:10.1371/journal.pone.0056550)
Supplement: Table S6 — Number of studies providing various types of ARV prophylaxis. (DOCX) [file pone.0056550.s007.docx]

**Table S6: Number of studies providing various types of ARV prophylaxis**

| ARV prophylaxis regimen: | | Number of studies: |
| --- | --- | --- |
| sdNVP | 24 | |
| ZDV | 6 | |
| ZDV or sdNVP | 4 | |
| ZDV and sdNVP | 1 | |
| ZDV and/or sdNVP | 1 | |
| ZDV/3TC/NFV | 1 | |
| ZDV/3TC/NVP | 2 | |
| ZDV or ZDV/NVP or HAART | 1 | |

Abbreviations: ARV, antiretroviral; HAART, highly active antiretroviral therapy; NFV, nelfinavir; NVP, nevirapine; sdNVP, single-dose nevirapine; ZDV, zidovudine; 3TC=lamivudine.
