# Supplementary material for: The Uptake of Integrated Perinatal Prevention of Mother-to-Child HIV Transmission Programs in Low- and Middle-Income Countries: A Systematic Review
Source: PLoS One. 2013 Mar 6;8(3):e56550. doi: 10.1371/journal.pone.0056550 (PMC3590218; doi:10.1371/journal.pone.0056550)
Supplement: Text S1 — The World Health Organization definition of PMTCT program. (DOCX) [file pone.0056550.s008.docx]

Text S1: The World Health Organization definition of PMTCT programs

PMTCT interventions are divided into four prongs:

- primary prevention of HIV infection in women,
- prevention of unintended pregnancy among HIV-positive women,
- reduction of vertical transmission from HIV infected pregnant and lactating women to their children,
- care and support of women, infants, and families infected and affected by HIV/AIDS [[1](#_ENREF_1)].

1. WHO (2010) PMTCT strategic vision 2010-2015: preventing mother-to-child transmission of HIV to reach the UNGASS and Millennium Development Goals. Geneva: WHO.
