# Supplementary material for: The Uptake of Integrated Perinatal Prevention of Mother-to-Child HIV Transmission Programs in Low- and Middle-Income Countries: A Systematic Review
Source: PLoS One. 2013 Mar 6;8(3):e56550. doi: 10.1371/journal.pone.0056550 (PMC3590218; doi:10.1371/journal.pone.0056550)
Supplement: Text S2 — List of primary and secondary outcomes. (DOCX) [file pone.0056550.s009.docx]

Text S2: List of primary and secondary outcomes

**Primary outcomes:**

- Proportion of pregnant women tested for HIV
- Proportion of HIV positive eligible pregnant women provided with ART (started as part of PMTCT)
- Proportion of HIV positive pregnant women provided with any ARV prophylaxis (preferably proportion of participants taking the full regimen according to the guidelines used in each study. Where this was not available, we included data on participants starting the ARV prophylaxis)
- Proportion of HIV positive pregnant women having a safe delivery (Safe delivery consist of delivery methods which aim to minimize the risk of transmission of HIV from mother to child during the delivery. These practices include elective caesarean section if in hospital or vaginal delivery with minimal use of the invasive procedures, administration of antiretroviral prophylaxis to mother on the onset of labor and to the infant after delivery and presence of a trained homebirth attendant at home births).
- Proportion of infants born to HIV positive women provided with ARV prophylaxis
- Proportion of HIV positive women provided with infant feeding counseling
- Proportion of infants born to HIV positive women tested for HIV

**Secondary outcomes:**

- The proportion of HIV negative infants born to HIV positive women
- The analysis of costs of integrated PMTCT programs
- The impact on human resources (i.e. increase/decrease in staff workload, number of health workers leaving/entering the services, increase/decrease in motivation of the health workers, accidental HIV infection among health workers)
- The quality of care (i.e. interpersonal communication, confidentiality, adequate counseling, infection prevention)
- Stigma (did the participants experience the stigma, the forms of stigma experienced, by whom was stigma manifested, did it cause higher attrition rates)
- Inequalities in access to healthcare services
- Barriers to implementation and sustainability of integrated programs
- The impact on attendance of healthcare services integrated with PMTCT programs
- The follow-up of HIV positive women and their children after the delivery i.e. provision of ART and co-trimoxazole to HIV positive women and HIV positive infants after the delivery
