# Supplementary material for: The Uptake of Integrated Perinatal Prevention of Mother-to-Child HIV Transmission Programs in Low- and Middle-Income Countries: A Systematic Review
Source: PLoS One. 2013 Mar 6;8(3):e56550. doi: 10.1371/journal.pone.0056550 (PMC3590218; doi:10.1371/journal.pone.0056550)
Supplement: Text S3 — Exclusion criteria. (DOCX) [file pone.0056550.s010.docx]

**Text S3: Exclusion criteria**

We excluded studies not reporting on primary outcomes, studies without description of integrated care and studies reporting on integration of only one perinatal PMTCT intervention since we looked at these programs holistically and our focus was on the loss to follow-up between each step of the PMTCT program. We also excluded studies about patients’ acceptability of PMTCT services and studies presenting pooled outcomes of integrated PMTCT programs from more than one country since different countries have different challenges and contexts of integrated care. Studies retrospectively analyzing PMTCT interventions uptake were also excluded.
