# Supplementary material for: The Uptake of Integrated Perinatal Prevention of Mother-to-Child HIV Transmission Programs in Low- and Middle-Income Countries: A Systematic Review
Source: PLoS One. 2013 Mar 6;8(3):e56550. doi: 10.1371/journal.pone.0056550 (PMC3590218; doi:10.1371/journal.pone.0056550)
Supplement: Text S4 — Search strategy. (DOCX) [file pone.0056550.s011.docx]

**Text S4: Search strategy**

Box 1: Search Strategy for AIDS Education Global Information System (AEGIS) (27 July 2010)

| (mother-to-child OR MTCT OR mother to child OR PMTCT OR postnatal transmission OR prenatal transmission OR vertical transmission OR HIV transmission OR mother-to-infant OR maternal-to-child OR maternal-infant) |
| --- |

Box 2: Search Strategy for British Library Catalogue (BETA) (18 August 2010)

| (mother-to-child OR MTCT OR mother-to-infant OR adult-to-child OR maternal-to-child OR vertical transmission OR perinatal transmission OR postnatal transmission OR post natal transmission OR maternal-infant transmission OR PMTCT) AND (HIV OR AIDS) |
| --- |

Box 3: Search Strategy for Cumulative Index to Nursing and Allied Health Literature (CINAHL) (25 July 2010)

| ("mother-to-child" OR MTCT OR "mother to child" OR "mother-to-infant" OR "adult-to-child" OR "maternal-to-child" OR "vertical transmission" OR "perinatal transmission" OR "prenatal transmission" OR "postnatal transmission" OR "post natal transmission" OR "maternal-infant transmission" OR PMTCT) and (HIV OR HIV-1* OR HIV-2* OR HIV-1 OR HIV2 OR "HIV infect*" OR "human immunodeficiency virus" OR "human immunodeficiency virus" OR "human immune-deficiency virus" OR "human immuno-deficiency virus" OR "human immun* deficiency virus" OR aids OR "acquired immunodeficiency syndrome" OR "acquired immunodeficiency syndrome" OR "acquired immuno-deficiency syndrome" OR "acquired immune-deficiency syndrome" OR "acquired immun* deficiency syndrome") |
| --- |

Box 4: Search Strategy for CLINICALTRIALS.gov (28 July 2010)

| mother-to-child OR MTCT OR mother to child OR PMTCT OR postnatal transmission OR prenatal transmission OR vertical transmission OR HIV transmission OR mother-to-infant OR maternal-to-child OR maternal-infant \| HIV OR HIV/AIDS |
| --- |

Box 5: Search Strategy for [Cochrane Central Register of Controlled Trials (CENTRAL)](http://www.mrw.interscience.wiley.com/cochrane/cochrane_clcentral_articles_fs.html) (26 July 2010)

| #1[MeSH descriptor HIV Infections explode all trees](http://www3.interscience.wiley.com/cochrane/searchHistory?mode=runquery&qnum=1)  #2[MeSH descriptor HIV explode all trees](http://www3.interscience.wiley.com/cochrane/searchHistory?mode=runquery&qnum=2)  [hiv OR hiv-1* OR hiv-2* OR hiv1 OR hiv2 OR HIV INFECT* OR HUMAN IMMUNODEFICIENCY VIRUS OR HUMAN IMMUNEDEFICIENCY VIRUS OR HUMAN IMMUNE-DEFICIENCY VIRUS OR HUMAN IMMUNO-DEFICIENCY VIRUS OR HUMAN IMMUN* DEFICIENCY VIRUS OR ACQUIRED IMMUNODEFICIENCY SYNDROME OR ACQUIRED IMMUNEDEFICIENCY SYNDROME OR ACQUIRED IMMUNO-DEFICIENCY SYNDROME OR ACQUIRED IMMUNE-DEFICIENCY SYNDROME OR ACQUIRED IMMUN* DEFICIENCY SYNDROME](http://www3.interscience.wiley.com/cochrane/searchHistory?mode=runquery&qnum=3)  #4[MeSH descriptor Lymphoma, AIDS-Related, this term only](http://www3.interscience.wiley.com/cochrane/searchHistory?mode=runquery&qnum=4)  #5[MeSH descriptor Sexually Transmitted Diseases, Vira**l**, this term only](http://www3.interscience.wiley.com/cochrane/searchHistory?mode=runquery&qnum=5)  #6[(#1 OR #2 OR #3 OR #4 OR #5)](http://www3.interscience.wiley.com/cochrane/searchHistory?mode=runquery&qnum=6)  #7[MeSH descriptorInfectious Disease Transmission, Vertical**,** this term only](http://www3.interscience.wiley.com/cochrane/searchHistory?mode=runquery&qnum=7)  #8[mother-to-child OR MTCT OR mother-to-infant OR adult-to-child OR maternal-to-child OR vertical transmission OR perinatal transmission OR postnatal transmission OR post natal transmission OR maternal-infant transmission OR PMTCT](http://www3.interscience.wiley.com/cochrane/searchHistory?mode=runquery&qnum=8)  #9[(#7 OR #8)](http://www3.interscience.wiley.com/cochrane/searchHistory?mode=runquery&qnum=9)  #10[(#6 AND #9), from 1990 to 2010](http://www3.interscience.wiley.com/cochrane/searchHistory?mode=runquery&qnum=10) |
| --- |

Box 6: Search Strategy for Cochrane Database of Systematic Reviews (26 July 2010)

| #1[MeSH descriptor HIV Infections explode all trees](http://www3.interscience.wiley.com/cochrane/searchHistory?mode=runquery&qnum=1)  #2[MeSH descriptorHIVexplode all trees](http://www3.interscience.wiley.com/cochrane/searchHistory?mode=runquery&qnum=2)  #3hiv OR hiv-1* OR hiv-2* OR hiv1 OR hiv2 OR HIV INFECT* OR HUMAN IMMUNODEFICIENCY VIRUS OR HUMAN IMMUNEDEFICIENCY VIRUS OR HUMAN IMMUNE-DEFICIENCY VIRUS OR HUMAN IMMUNO-DEFICIENCY VIRUS OR HUMAN IMMUN* DEFICIENCY VIRUS OR ACQUIRED IMMUNODEFICIENCY SYNDROME OR ACQUIRED IMMUNEDEFICIENCY SYNDROME OR ACQUIRED IMMUNO-DEFICIENCY SYNDROME OR ACQUIRED IMMUNE-DEFICIENCY SYNDROME OR ACQUIRED IMMUN* DEFICIENCY SYNDROME  #4[MeSH descriptorLymphoma, AIDS-Related**,** this term only](http://www3.interscience.wiley.com/cochrane/searchHistory?mode=runquery&qnum=4)  #5[MeSH descriptor Sexually Transmitted Diseases, Viral, this term only](http://www3.interscience.wiley.com/cochrane/searchHistory?mode=runquery&qnum=5)  #6[(#1 OR #2 OR #3 OR #4 OR #5)](http://www3.interscience.wiley.com/cochrane/searchHistory?mode=runquery&qnum=6)  #7[MeSH descriptorInfectious Disease Transmission, Vertical**,** this term only](http://www3.interscience.wiley.com/cochrane/searchHistory?mode=runquery&qnum=7)  #8[mother-to-child OR MTCT OR mother-to-infant OR adult-to-child OR maternal-to-child OR vertical transmission OR perinatal transmission OR postnatal transmission OR post natal transmission OR maternal-infant transmission OR PMTCT](http://www3.interscience.wiley.com/cochrane/searchHistory?mode=runquery&qnum=8)  #9[(#7 OR #8)](http://www3.interscience.wiley.com/cochrane/searchHistory?mode=runquery&qnum=9)  #10[(#6 AND #9), from 1990 to 2010](http://www3.interscience.wiley.com/cochrane/searchHistory?mode=runquery&qnum=10) |
| --- |

**Box 7: Search Strategy for Database of Abstracts of Reviews on Effects (26 July 2010)**

| #1[MeSH descriptor HIV Infections explode all trees](http://www3.interscience.wiley.com/cochrane/searchHistory?mode=runquery&qnum=1)  #2[MeSH descriptor HIV explode all trees](http://www3.interscience.wiley.com/cochrane/searchHistory?mode=runquery&qnum=2)  #3[HIV OR HIV-1* OR HIV-2* OR HIV1 OR HIV2 OR HIV INFECT* OR HUMAN IMMUNODEFICIENCY VIRUS OR HUMAN IMMUNEDEFICIENCY VIRUS OR HUMAN IMMUNE-DEFICIENCY VIRUS OR HUMAN IMMUNO-DEFICIENCY VIRUS OR HUMAN IMMUN* DEFICIENCY VIRUS OR ACQUIRED IMMUNODEFICIENCY SYNDROME OR ACQUIRED IMMUNEDEFICIENCY SYNDROME OR ACQUIRED IMMUNO-DEFICIENCY SYNDROME OR ACQUIRED IMMUNE-DEFICIENCY SYNDROME OR ACQUIRED IMMUN* DEFICIENCY SYNDROME](http://www3.interscience.wiley.com/cochrane/searchHistory?mode=runquery&qnum=3)  #4[MeSH descriptor Lymphoma, AIDS-Related, this term only](http://www3.interscience.wiley.com/cochrane/searchHistory?mode=runquery&qnum=4)  #5[MeSH descriptor Sexually Transmitted Diseases, Viral**,** this term only](http://www3.interscience.wiley.com/cochrane/searchHistory?mode=runquery&qnum=5)  #6[(#1 OR #2 OR #3 OR #4 OR #5)](http://www3.interscience.wiley.com/cochrane/searchHistory?mode=runquery&qnum=6)  #7[MeSH descriptorInfectious Disease Transmission, Vertical**,** this term only](http://www3.interscience.wiley.com/cochrane/searchHistory?mode=runquery&qnum=7)  #8mother-to-child OR MTCT OR mother-to-infant OR adult-to-child OR maternal-to-child OR vertical transmission OR perinatal transmission OR postnatal transmission OR post natal transmission OR maternal-infant transmission OR PMTCT  #9[(#7 OR #8)](http://www3.interscience.wiley.com/cochrane/searchHistory?mode=runquery&qnum=9)  #10[(#6 AND #9), from 1990 to 2010](http://www3.interscience.wiley.com/cochrane/searchHistory?mode=runquery&qnum=10) |
| --- |

**Box 8: Search strategy for Education Resources Information Center (ERIC) (18 August 2010)**

| (mother-to-child OR MTCT OR mother-to-infant OR adult-to-child OR maternal-to-child OR vertical transmission OR perinatal transmission OR postnatal transmission OR post natal transmission OR maternal-infant transmission OR PMTCT) AND (HIV OR AIDS) |
| --- |

**Box 9: Search Strategy for EMBASE (26 July 2010)**

| #1 'human immunodeficiency virus infection'/exp OR'human immunodeficiency virus infection'OR 'human immunodeficiency virus'/exp OR'human immunodeficiency virus'OR 'b cell lymphoma'/de OR'b cell lymphoma'OR hiv:ti ORhiv**:**ab OR'hiv-1':ti OR'hiv-1'**:**ab OR'hiv-2'**:**ti OR'hiv-2'**:**ab OR'human immunodeficiency virus'**:**ti OR'human immunodeficiency virus'**:**ab OR'human immunedeficiency virus'**:**ti OR'human immunedeficiency virus'**:**ab OR'human immune-deficiency virus':ti OR'human immune-deficiency virus'**:**ab OR'human immuno-deficiency virus':ti OR'human immuno-deficiency virus':ab OR'acquired immunodeficiency syndrome':ti OR'acquired immunodeficiency syndrome':ab OR'acquired immuno-deficiency syndrome':ti OR 'acquired immuno-deficiency syndrome':ab OR'acquired immune-deficiency syndrome':ti OR'acquired immune-deficiency syndrome':ab OR'acquired immunedeficiency syndrome':ti OR'acquired immunedeficiency syndrome'**:**ab  #2 'mother-to-child transmission' ORmtct OR'mother-to-infant' OR'adult-to-child' OR'maternal-to-child' OR'vertical transmission'/de OR'vertical transmission'OR'perinatal transmission' OR'postnatal transmission' OR'post natal transmission' OR'maternal-infant transmission'ORpmtct OR'disease transmission, vertical'**/**de OR'disease transmission, vertical'  #3 #1 AND #2  #4 #1 AND #2AND [humans]/lim AND [embase]/lim AND [1990-2010]/py |
| --- |

**Box 10: Search Strategy for Global Health (CAB Abstracts) (27 August 2010)**

| Topic = (mother-to-child OR MTCT OR mother-to-infant OR adult-to-child OR maternal-to-child OR vertical transmission OR perinatal transmission OR postnatal transmission OR post natal transmission OR maternal-infant  AND  Topic = (HIV OR hiv-1* OR HIV-2* OR HIV1 OR HIV2 OR HIV infect* OR human immunodeficiency virus OR human immunedeficiency virus OR human immune-deficiency virus OR human immuno-deficiency virus OR human immun* deficiency virus OR acquired immunodeficiency syndrome OR acquired immunedeficiency syndrome OR acquired immuno-deficiency syndrome OR acquired immune-deficiency syndrome OR acquired immun* deficiency syndrome transmission OR PMTCT) |
| --- |

**Box 11: Search Strategy for Google Scholar (11 August 2010)**

| With at least one of the words: "mother to child" MTCT PMTCT "postnatal transmission" "prenatal transmission" "vertical transmission" "HIV transmission" "mother to infant" "maternal to child" "maternal-infant" |
| --- |

**Box 12: Search Strategy for MEDLINE (26 July 2010)**

| #1 Search HIV Infections[MeSH] OR HIV[MeSH] OR hiv[tw] OR hiv-1*[tw] OR hiv-2*[tw] OR hiv1[tw] OR hiv2[tw] OR hiv infect*[tw] OR human immunodeficiency virus[tw] OR human immunedeficiency virus[tw] OR human immuno-deficiency virus[tw] OR human immune-deficiency virus[tw] OR ((human immun*) AND (deficiency virus[tw])) OR acquired immunodeficiency syndrome[tw] OR acquired immunedeficiency syndrome[tw] OR acquired immuno-deficiency syndrome[tw] OR acquired immune-deficiency syndrome[tw] OR ((acquired immun*) AND (deficiency syndrome[tw])) OR "sexually transmitted diseases, viral:noexp"[MH]  [#](http://www.ncbi.nlm.nih.gov/pubmed/advanced?querykey=5&dbase=pubmed&querytype=eSearch&)2 Search mother-to-child[tiab] OR MTCT[tiab] OR mother-to-infant[tiab] OR adult-to-child[tiab] OR maternal-to-child[tiab] OR vertical transmission[tiab] OR perinatal transmission[tiab] OR postnatal transmission[tiab] OR post natal transmission[tiab] OR maternal-infant transmission[tiab] OR PMTCT[tiab] OR infectious disease transmission, vertical/prevention and control[mh]  [#](http://www.ncbi.nlm.nih.gov/pubmed/advanced?querykey=6&dbase=pubmed&querytype=eSearch&)3Search #4 AND #5  [#](http://www.ncbi.nlm.nih.gov/pubmed/advanced?querykey=7&dbase=pubmed&querytype=eSearch&)4 Search (#4 AND #5) NOT (animals[mh] NOT humans[mh])  [#](http://www.ncbi.nlm.nih.gov/pubmed/advanced?querykey=8&dbase=pubmed&querytype=eSearch&)5 Search (#4 AND #5) NOT (animals[mh] NOT humans[mh]) Limits: Publication Date from 1990/01/01 to 2010/07/26 |
| --- |

**Box 13: Search Strategy for New York Academy of Medicine Grey Literature Collection (12 August 2010)**

| Mother-to-child  “mother-to-child” returned 172 results.  “kw,wrdl: mother-to-child and kw,wrdl: hiv yr,st-numeric,ge=1990 and yr,st-numeric,le=2010” returned 23 results  Mother to child  “Mother to child” returned 319 results  “kw,wrdl: mother to child and kw,wrdl: hiv yr,st-numeric,ge=1990 and yr,st-numeric,le=2010” returned 45 results  MTCT  “mtct ” returned 5 results  “kw,wrdl: mtct yr,st-numeric,ge=1990 and yr,st-numeric,le=2010” returned 2 results  PMTCT  “pmtct ” returned 2 results  “kw,wrdl: pmtct yr,st-numeric,ge=1990 and yr,st-numeric,le=2010” returned 2 results  Postnatal transmission  No results match your search for “postnatal transmission” in The New York Academy of Medicine Library Catalog  Prenatal transmission  “prenatal transmission ” returned 2 results  “kw,wrdl: prenatal transmission yr,st-numeric,ge=1990 and yr,st-numeric,le=2010” returned 2 results  Vertical transmission  “Vertical transmission” returned 7 results  “kw,wrdl: vertical transmission yr,st-numeric,ge=1990 and yr,st-numeric,le=2010” returned 7 results  HIV transmission  “hiv transmission ” returned 89 results  “kw,wrdl: hiv transmission yr,st-numeric,ge=1990 and yr,st-numeric,le=2010” returned 69 results  Mother-to-infant  “mother-to-infant” returned 63 results  “kw,wrdl: mother-to-infant and kw,wrdl: hiv yr,st-numeric,ge=1990 and yr,st-numeric,le=2010” returned 7 results  Maternal-to-child  “maternal-to-child” returned 132 results  “kw,wrdl: maternal-to-child and kw,wrdl: hiv yr,st-numeric,ge=1990 and yr,st-numeric,le=2010” returned 20 results |
| --- |

**Box 14: Search Strategy for OpenSIGLE (11 August 2010)**

| ("mother-to-child" OR MTCT OR "mother to child" OR PMTCT OR "postnatal transmission" OR "prenataltransmission" OR vertical transmission OR transmission OR "mother-to-infant" OR "maternal-to-child" OR"maternal-infant") AND (HIV OR AIDS) |
| --- |

**Box 15: Search Strategy for Population Information Online (POPLINE) (18 August 2010)**

| TITLE/KEYWORDS (mother to child transmission / MTCT / prevention of mother to child transmission / PMTCT / postnatal transmission / prenatal transmission / perinatal transmission / vertical transmission / HIV transmission / mother-to-infant / maternal-to-child / maternal-infant) & (HIV/AIDS) |
| --- |

**Box 16: Search Strategy for ProQuest Dissertations and Theses Database (11 August 2010)**

| (mother-to-child OR MTCT OR mother to child OR PMTCT OR postnatal transmission OR prenatal transmission OR vertical transmission OR mother-to-infant OR maternal-to-child OR maternal-infant) AND (HIV OR AIDS) |
| --- |

| (HIV OR HIV-1* OR HIV-2* OR HIV1 OR HIV2 OR HIV infect* OR human immunodeficiency virus OR human immunedeficiency virus OR human immune-deficiency virus OR human immuno-deficiency virus OR human immun* deficiency virus OR acquired immunodeficiency syndrome OR acquired immunedeficiency syndrome OR acquired immuno-deficiency syndrome OR acquired immune-deficiency syndrome OR acquired immun* deficiency syndrome) AND ([mother-to-child OR MTCT OR mother-to-infant OR adult-to-child OR maternal-to-child OR vertical transmission OR perinatal transmission OR postnatal transmission OR post natal transmission OR maternal-infant transmission OR PMTCT](http://www3.interscience.wiley.com/cochrane/searchHistory?mode=runquery&qnum=8)) |
| --- |

**Box 17: Search Strategy for PsychINFO (27 August 2010)**

**Box 18: Search Strategy for Sociological Abstracts (18 August 2010)**

| (mother-to-child OR MTCT OR mother-to-infant OR adult-to-child OR maternal-to-child OR vertical transmission OR perinatal transmission OR postnatal transmission OR post natal transmission OR maternal-infant transmission OR PMTCT) AND (HIV OR AIDS) |
| --- |

**Box 19: Search Strategy 1 (1990 - 2000) for U.S. National Library of Medicine’s (NLM) Gateway system (3August 2010)**

| #1 ("HIV Infections"[MeSH] OR "HIV"[MeSH] OR hiv[tw] OR hiv-1*[tw] OR hiv-2*[tw] OR hiv1[tw] OR hiv2[tw] OR hiv infect*[tw] OR human immunodeficiency virus[tw] OR human immunedeficiency virus[tw] OR human immuno-deficiency virus[tw] OR human immune-deficiency virus[tw]) OR (((human immun*) AND (deficiency virus[tw])) OR acquired immunodeficiency syndrome[tw] OR acquired immunedeficiency syndrome[tw] OR acquired immuno-deficiency syndrome[tw] OR acquired immune-deficiency syndrome[tw] OR ((acquired immun*) AND (deficiency syndrome[tw])) OR "Sexually Transmitted Diseases, Viral"[MeSH:NoExp])  #2Search mother-to-child[tw] OR MTCT[tw] OR mother-to-infant[tw] OR adult-to-child[tw] OR maternal-to-child[tw] OR vertical transmission[tw] OR perinatal transmission[tw] OR postnatal transmission[tw] OR post natal transmission[tw] OR maternal-infant transmission[tw] OR PMTCT[tw] OR infectious disease transmission, vertical/prevention and control[mh] #3 Search: ((("HIV Infections"[MeSH] OR "HIV"[MeSH] OR hiv[tw] OR hiv-1*[tw] OR hiv-2*[tw] OR hiv1[tw] OR hiv2[tw] OR hiv infect*[tw] OR human immunodeficiency virus[tw] OR human immunedeficiency virus[tw] OR human immuno-deficiency virus[tw] OR human immune-deficiency virus[tw]) OR (((human immun*) AND (deficiency virus[tw])) OR acquired immunodeficiency syndrome[tw] OR acquired immunedeficiency syndrome[tw] OR acquired immuno-deficiency syndrome[tw] OR acquired immune-deficiency syndrome[tw] OR ((acquired immun*) AND (deficiency syndrome[tw])) OR "Sexually Transmitted Diseases, Viral"[MeSH:NoExp])) AND (Search mother-to-child[tw] OR MTCT[tw] OR mother-to-infant[tw] OR adult-to-child[tw] OR maternal-to-child[tw] OR vertical transmission[tw] OR perinatal transmission[tw] OR postnatal transmission[tw] OR post natal transmission[tw] OR maternal-infant transmission[tw] OR PMTCT[tw] OR infectious disease transmission, vertical/prevention and control[mh])) NOT (animals[mh] NOT humans[mh]) Limit: 1990/01/01:2000/12/31  Search Strategy 2 (2001 - 2005)  #1("HIV Infections"[MeSH] OR "HIV"[MeSH] OR hiv[tw] OR hiv-1*[tw] OR hiv-2*[tw] OR hiv1[tw] OR hiv2[tw] OR hiv infect*[tw] OR human immunodeficiency virus[tw] OR human immunedeficiency virus[tw] OR human immuno-deficiency virus[tw] OR human immune-deficiency virus[tw]) OR (((human immun*) AND (deficiency virus[tw])) OR acquired immunodeficiency syndrome[tw] OR acquired immunedeficiency syndrome[tw] OR acquired immuno-deficiency syndrome[tw] OR acquired immune-deficiency syndrome[tw] OR ((acquired immun*) AND (deficiency syndrome[tw])) OR "Sexually Transmitted Diseases, Viral"[MeSH:NoExp])  #2Search mother-to-child[tw] OR MTCT[tw] OR mother-to-infant[tw] OR adult-to-child[tw] OR maternal-to-child[tw] OR vertical transmission[tw] OR perinatal transmission[tw] OR postnatal transmission[tw] OR post natal transmission[tw] OR maternal-infant transmission[tw] OR PMTCT[tw] OR infectious disease transmission, vertical/prevention and control[mh]  #3 ((("HIV Infections"[MeSH] OR "HIV"[MeSH] OR hiv[tw] OR hiv-1*[tw] OR hiv-2*[tw] OR hiv1[tw] OR hiv2[tw] OR hiv infect*[tw] OR human immunodeficiency virus[tw] OR human immunedeficiency virus[tw] OR human immuno-deficiency virus[tw] OR human immune-deficiency virus[tw]) OR (((human immun*) AND (deficiency virus[tw])) OR acquired immunodeficiency syndrome[tw] OR acquired immunedeficiency syndrome[tw] OR acquired immuno-deficiency syndrome[tw] OR acquired immune-deficiency syndrome[tw] OR ((acquired immun*) AND (deficiency syndrome[tw])) OR "Sexually Transmitted Diseases, Viral"[MeSH:NoExp])) AND (Search mother-to-child[tw] OR MTCT[tw] OR mother-to-infant[tw] OR adult-to-child[tw] OR maternal-to-child[tw] OR vertical transmission[tw] OR perinatal transmission[tw] OR postnatal transmission[tw] OR post natal transmission[tw] OR maternal-infant transmission[tw] OR PMTCT[tw] OR infectious disease transmission, vertical/prevention and control[mh])) NOT (animals[mh] NOT humans[mh]) Limit: 2001/01/01:2005/12/31  Search Strategy 3 (2006 - 2010)  #1("HIV Infections"[MeSH] OR "HIV"[MeSH] OR hiv[tw] OR hiv-1*[tw] OR hiv-2*[tw] OR hiv1[tw] OR hiv2[tw] OR hiv infect*[tw] OR human immunodeficiency virus[tw] OR human immunedeficiency virus[tw] OR human immuno-deficiency virus[tw] OR human immune-deficiency virus[tw]) OR (((human immun*) AND (deficiency virus[tw])) OR acquired immunodeficiency syndrome[tw] OR acquired immunedeficiency syndrome[tw] OR acquired immuno-deficiency syndrome[tw] OR acquired immune-deficiency syndrome[tw] OR ((acquired immun*) AND (deficiency syndrome[tw])) OR "Sexually Transmitted Diseases, Viral"[MeSH:NoExp])  #2Search mother-to-child[tw] OR MTCT[tw] OR mother-to-infant[tw] OR adult-to-child[tw] OR maternal-to-child[tw] OR vertical transmission[tw] OR perinatal transmission[tw] OR postnatal transmission[tw] OR post natal transmission[tw] OR maternal-infant transmission[tw] OR PMTCT[tw] OR infectious disease transmission, vertical/prevention and control[mh]  #3 ((("HIV Infections"[MeSH] OR "HIV"[MeSH] OR hiv[tw] OR hiv-1*[tw] OR hiv-2*[tw] OR hiv1[tw] OR hiv2[tw] OR hiv infect*[tw] OR human immunodeficiency virus[tw] OR human immunedeficiency virus[tw] OR human immuno-deficiency virus[tw] OR human immune-deficiency virus[tw]) OR (((human immun*) AND (deficiency virus[tw])) OR acquired immunodeficiency syndrome[tw] OR acquired immunedeficiency syndrome[tw] OR acquired immuno-deficiency syndrome[tw] OR acquired immune-deficiency syndrome[tw] OR ((acquired immun*) AND (deficiency syndrome[tw])) OR "Sexually Transmitted Diseases, Viral"[MeSH:NoExp])) AND (Search mother-to-child[tw] OR MTCT[tw] OR mother-to-infant[tw] OR adult-to-child[tw] OR maternal-to-child[tw] OR vertical transmission[tw] OR perinatal transmission[tw] OR postnatal transmission[tw] OR post natal transmission[tw] OR maternal-infant transmission[tw] OR PMTCT[tw] OR infectious disease transmission, vertical/prevention and control[mh])) NOT (animals[mh] NOT humans[mh]) Limit: 2001/01/01:2005/12/31 |
| --- |

**Box 20: Search Strategy for WHO International Clinical Trials Registry Platform (WHO ICTRP) (27 July 2010)**

| (mother-to-child OR MTCT OR mother to child OR PMTCT OR postnatal transmission OR prenatal transmission OR vertical transmission OR HIV transmission OR mother-to-infant OR maternal-to-child OR maternal-infant) in **TITLE** AND (HIV OR HIV/AIDS) in **CONDITION**AND **DATE OF REGISTRATION** is between (01/01/1990 – 27/07/2010) |
| --- |

**Box 21: Search Strategy for World Health Organization’s The Global Health Library (18 August 2010)**

| (HIV OR AIDS) AND (mother-to-child OR MTCT OR mother to child OR PMTCT OR postnatal transmission OR prenatal transmission OR vertical transmission OR HIV transmission OR mother-to-infant OR maternal-to-child OR maternal-infant) |
| --- |
