# Supplementary material for: The Uptake of Integrated Perinatal Prevention of Mother-to-Child HIV Transmission Programs in Low- and Middle-Income Countries: A Systematic Review
Source: PLoS One. 2013 Mar 6;8(3):e56550. doi: 10.1371/journal.pone.0056550 (PMC3590218; doi:10.1371/journal.pone.0056550)
Supplement: Text S5 — Description of denominators. (DOCX) [file pone.0056550.s012.docx]

**Text S5: Description of denominators**

To obtain uniform and comparable data, we had to recalculate some of the outcomes reported in the included studies using predefined denominators. We used the total number of attendees at antenatal care service as the denominator to present the proportion of women who received information on PMTCT and who were tested for HIV at the antenatal care level. Studies which did not provide the information about the number of antenatal care attendees were not used in this analysis. To determine the proportion of women who collected the test results, we used the number of women tested for HIV as denominator. For each of the subsequent steps of the PMTCT programs, we used the number of women identified as HIV positive as denominator. In studies presenting PMTCT program delivered at labor ward, we used the number of women attending labor ward as denominator to calculate the proportion of women counseled and tested for HIV. To determine the proportion of women receiving the rest of the PMTCT interventions at labor ward, we used the number of identified HIV positive women at labor ward. To determine the proportion of infants provided with ARV prophylaxis we used the number of infants of HIV positive women born alive. To calculate the number of infants tested for HIV, we used the number of infants of HIV positive women born alive.
