# Supplementary material for: The Uptake of Integrated Perinatal Prevention of Mother-to-Child HIV Transmission Programs in Low- and Middle-Income Countries: A Systematic Review
Source: PLoS One. 2013 Mar 6;8(3):e56550. doi: 10.1371/journal.pone.0056550 (PMC3590218; doi:10.1371/journal.pone.0056550)
Supplement: Text S6 — Description of setting, participants and staff from the included studies. (DOCX) [file pone.0056550.s013.docx]

**Text S6: Description of setting, participants and staff from the included studies**

The PMTCT program was integrated across antenatal, labor ward and postnatal care in 24 studies [[1-24](#_ENREF_1)], in antenatal and postnatal care in two studies [[25](#_ENREF_25),[26](#_ENREF_26)], in antenatal and labor ward care in nine studies [[27-35](#_ENREF_27)], in antenatal care in one study [[36](#_ENREF_36)], at labor ward in four studies [[37-40](#_ENREF_37)], and in labor ward and postnatal care in one study [[41](#_ENREF_41)].

The antenatal care was provided in 15 studies at primary healthcare level [[1](#_ENREF_1),[2](#_ENREF_2),[4](#_ENREF_4),[7](#_ENREF_7),[9-11](#_ENREF_9),[13](#_ENREF_13),[17](#_ENREF_17),[21](#_ENREF_21),[23](#_ENREF_23),[28](#_ENREF_28),[32](#_ENREF_32),[33](#_ENREF_33),[36](#_ENREF_36)], while in 15 it occurred at hospital level [[3](#_ENREF_3),[6](#_ENREF_6),[8](#_ENREF_8),[12](#_ENREF_12),[14](#_ENREF_14),[16](#_ENREF_16),[18](#_ENREF_18),[19](#_ENREF_19),[24](#_ENREF_24),[26](#_ENREF_26),[29](#_ENREF_29),[31](#_ENREF_31),[34](#_ENREF_34),[35](#_ENREF_35),[39](#_ENREF_39)]. In four studies the location was not clear [[20](#_ENREF_20),[22](#_ENREF_22),[25](#_ENREF_25),[27](#_ENREF_27)]. The integrated PMTCT and labor ward care was always implemented at hospital level, except in one study where it was provided at primary healthcare level [[4](#_ENREF_4)].

The setting of care was not clearly reported in a number of studies. However, the setting was clearly reported as urban in 22 studies [[3](#_ENREF_3),[4](#_ENREF_4),[7](#_ENREF_7),[15-17](#_ENREF_15),[19](#_ENREF_19),[21](#_ENREF_21),[22](#_ENREF_22),[24-27](#_ENREF_24),[30-32](#_ENREF_30),[34-39](#_ENREF_34)], rural in nine studies [[12](#_ENREF_12),[14](#_ENREF_14),[23](#_ENREF_23),[29](#_ENREF_29),[33](#_ENREF_33)], and both, rural and urban in six studies [[1](#_ENREF_1),[5](#_ENREF_5),[9-11](#_ENREF_9),[13](#_ENREF_13)]. In one study integrated care was provided in a refugee camp [[2](#_ENREF_2)].

Only a few studies presented data on socio-demographic characteristics of the participants. In six studies the women were mostly unemployed or housewives [[2](#_ENREF_2),[11](#_ENREF_11),[13](#_ENREF_13),[14](#_ENREF_14),[16](#_ENREF_16),[41](#_ENREF_41)]. In seven studies most of the participants had primary level education or less [[5](#_ENREF_5),[13](#_ENREF_13),[14](#_ENREF_14),[17](#_ENREF_17),[25](#_ENREF_25),[28](#_ENREF_28),[41](#_ENREF_41)]. in three studies the majority of women finished secondary level education [[6](#_ENREF_6),[16](#_ENREF_16),[26](#_ENREF_26)], and in one study from Nigeria more than half of the women were college graduates [[34](#_ENREF_34)].

In all studies that reported on socio-demographic characteristics women were either married or in a relationship [[5-9](#_ENREF_5),[14](#_ENREF_14),[16](#_ENREF_16),[18](#_ENREF_18),[21](#_ENREF_21),[26](#_ENREF_26),[28](#_ENREF_28),[41](#_ENREF_41)].

In four studies some of the participants were intravenous drug users [[7](#_ENREF_7),[15](#_ENREF_15),[17](#_ENREF_17),[38](#_ENREF_38)].

In most of the studies, nurses, midwifes and doctors delivered care. Counselors, lay counselors, health workers or TBAs, were an integral part of the team of providers in 21 studies [[1](#_ENREF_1),[2](#_ENREF_2),[4](#_ENREF_4),[6](#_ENREF_6),[8](#_ENREF_8),[10](#_ENREF_10),[13](#_ENREF_13),[21](#_ENREF_21),[24-26](#_ENREF_24),[28](#_ENREF_28),[30-32](#_ENREF_30),[34](#_ENREF_34),[36](#_ENREF_36),[37](#_ENREF_37),[39-41](#_ENREF_39)].

In 25 studies the authors stated that the staff was trained to provide integrated care [[1](#_ENREF_1),[2](#_ENREF_2),[4-13](#_ENREF_4),[15](#_ENREF_15),[19-24](#_ENREF_19),[27](#_ENREF_27),[29](#_ENREF_29),[35](#_ENREF_35),[36](#_ENREF_36),[39](#_ENREF_39),[41](#_ENREF_41)].

1. Torpey K, Kabaso M, Kasonde P, Dirks R, Bweupe M, et al. (2010) Increasing the uptake of prevention of mother-to-child transmission of HIV services in a resource-limited setting. BMC Health ServRes 10: 29.

2. Rutta E, Gongo R, Mwansasu A, Mutasingwa D, Rwegasira V, et al. (2008) Prevention of mother-to-child transmission of HIV in a refugee camp setting in Tanzania. Glob Public Health 3: 62-76.

3. Viani RM, Ruiz-Calderon J, Lopez G, Chacon-Cruz E, Spector SA (2010) Mother-to-child HIV transmission in a cohort of pregnant women diagnosed by rapid HIV testing at Tijuana General Hospital, Baja California, Mexico. J Int Assoc Physicians AIDS Care (ChicIll) 9: 82-86.

4. Abdullah MF, Young T, Bitalo L, Coetzee N, Myers JE (2001) Public health lessons from a pilot programme to reduce mother-to-child transmission of HIV-1 in Khayelitsha. S Afr Med J 91: 579-583.

5. Kanshana S, Thewanda D, Teeraratkul A, Limpakarnjanarat K, Amornwichet P, et al. (2000) Implementing short-course zidovudine to reduce mother-infant HIV transmission in a large pilot program in Thailand. AIDS 14: 1617-1623.

6. Perez F, Mukotekwa T, Miller A, Orne-Gliemann J, Glenshaw M, et al. (2004) Implementing a rural programme of prevention of mother-to-child transmission of HIV in Zimbabwe: first 18 months of experience. Trop Med Int Health 9: 774-783.

7. Malyuta R, Newell ML, Ostergren M, Thorne C, Zhilka N (2006) Prevention of mother-to-child transmission of HIV infection: Ukraine experience to date. Eur J Public Health 16: 123-127.

8. Manzi M, Zachariah R, Teck R, Buhendwa L, Kazima J, et al. (2005) High acceptability of voluntary counselling and HIV-testing but unacceptable loss to follow up in a prevention of mother-to-child HIV transmission programme in rural Malawi: scaling-up requires a different way of acting. Trop Med Int Health 10: 1242-1250.

9. Welty TK, Bulterys M, Welty ER, Tih PM, Ndikintum G, et al. (2005) Integrating prevention of mother-to-child HIV transmission into routine antenatal care: the key to program expansion in Cameroon. J Acquir Immune Defic Syndr 40: 486-493.

10. Doherty TM, McCoy D, Donohue S (2005) Health system constraints to optimal coverage of the prevention of mother-to-child HIV transmission programme in South Africa: lessons from the implementation of the national pilot programme. Afr Health Sci 5: 213-218.

11. Garcia R, Prieto F, Arenas C, Rincon J, Caicedo S, et al. (2005) [Reduction of HIV mother-to-child transmission in Colombia, two years of experience, 2003-2005]. Biomedica 25: 547-564.

12. Homsy J, Kalamya JN, Obonyo J, Ojwang J, Mugumya R, et al. (2006) Routine intrapartum HIV counseling and testing for prevention of mother-to-child transmission of HIV in a rural Ugandan hospital. J Acquir Immune Defic Syndr 42: 149-154.

13. Karcher H, Kunz A, Poggensee G, Mbezi P, Mugenyi K, et al. (2006) Outcome of Different Nevirapine Administration Strategies in Preventin g Mother-to-Child Transmission (PMTCT) Programs in Tanzania and Uganda. J Int AIDS Soc 8: 12.

14. Kasenga F, Hurtig AK, Emmelin M (2007) Home deliveries: implications for adherence to nevirapine in a PMTCT programme in rural Malawi. AIDS Care 19: 646-652.

15. Kissin DM, Akatova N, Rakhmanova AG, Vinogradova EN, Voronin EE, et al. (2008) Rapid HIV testing and prevention of perinatal HIV transmission in high-risk maternity hospitals in St. Petersburg, Russia. Am J Obstet Gynecol 198: 183-187.

16. Kouam L, Nsangou I, Mbanya D, Nkam M, Kongnyuy EJ, et al. (2006) Prevention of mother-to-child transmission of HIV in Cameroon: experiences from the University Teaching Hospital in Yaounde (Cameroon). Zentralbl Gynakol 128: 82-86.

17. Miranda AE, Soares RA, Prado BC, Monteiro RB, Figueiredo NC (2005) Mother to child transmission of HIV in Vitoria, Brazil: factors associated with lack of HIV prevention. AIDS Care 17: 721-728.

18. Parameshwari S, Jacob MS, Vijayakumari J, Shalini D, Sushi MK, et al. (2009) A Program on Prevention of Mother to Child Transmission of HIV at Government Hospital, Tiruchengode Taluk, Namakkal District. Indian J Community Med 34: 261-263.

19. Saman M, Kruy LS, Glaziou P, Rekacewicz C, Leng C, et al. (2002) Feasibility of antenatal and late HIV testing in pregnant women in Phnom Penh Cambodia: the PERIKAM/ANRS1205 study. AIDS 16: 950-951.

20. Saraceni V, Rapparini C, Fonseca AF, Lima KR, Israel G, et al. Prevention of mother to child HIV transmission - a public health matter in Rio de Janeiro City; 2000; Durban, South Africa.

21. Shetty AK, Marangwanda C, Stranix-Chibanda L, Chandisarewa W, Chirapa E, et al. (2008) The feasibility of preventing mother-to-child transmission of HIV using peer counselors in Zimbabwe. AIDS Res Ther 5: 17.

22. Stringer EM, Sinkala M, Stringer JS, Mzyece E, Makuka I, et al. (2003) Prevention of mother-to-child transmission of HIV in Africa: successes and challenges in scaling-up a nevirapine-based program in Lusaka, Zambia. AIDS 17: 1377-1382.

23. Wanyu B, Diom E, Mitchell P, Tih PM, Meyer DJ (2007) Birth attendants trained in "Prevention of Mother-To-Child HIV Transmission" provide care in rural Cameroon, Africa. J Midwifery Womens Health 52: 334-341.

24. Geddes R, Knight S, Reid S, Giddy J, Esterhuizen T, et al. (2008) Prevention of mother-to-child transmission of HIV programme: low vertical transmission in KwaZulu-Natal, South Africa. S Afr Med J 98: 458-462.

25. Deschamps MM, Noel F, Bonhomme J, Devieux JG, Saint-Jean G, et al. (2009) Prevention of mother-to-child transmission of HIV in Haiti. Rev Panam Salud Publica 25: 24-30.

26. Magoni M, Okong P, Bassani L, Kituka NP, Onyango S, et al. (2007) Implementation of a programme for the prevention of mother-to-child transmission of HIV in a Ugandan hospital over five years: challenges, improvements and lessons learned. Int J STD AIDS 18: 109-113.

27. Behets F, Mutombo GM, Edmonds A, Dulli L, Belting MT, et al. (2009) Reducing vertical HIV transmission in Kinshasa, Democratic Republic of Congo: trends in HIV prevalence and service delivery. AIDS Care 21: 583-590.

28. Ekouevi DK, Leroy V, Viho I, Bequet L, Horo A, et al. (2004) Acceptability and uptake of a package to prevent mother-to-child transmission using rapid HIV testing in Abidjan, Cote d'Ivoire. AIDS 18: 697-700.

29. Kirere MM, Sondag-Thull D, Lepage P (2008) Feasibility of prevention of perinatal HIV infection by nevirapine in rural areas of the northeast Democratic Republic of Congo, 2002-2004. J Med Virol 80: 772-776.

30. Le CT, Vu TT, Luu MC, Do TN, Dinh TH, et al. (2008) Preventing mother-to-child transmission of HIV in Vietnam: an assessment of progress and future directions. J Trop Pediatr 54: 225-232.

31. Moth IA, Ayayo ABCO, Kaseje DO (2005) Assessment of utilisation of PMTCT services at Nyanza Provincial Hospital, Kenya. SAHARA J 2: 244-250.

32. Msellati P, Hingst G, Kaba F, Viho I, Welffens-Ekra C, et al. (2001) Operational issues in preventing mother-to-child transmission of HIV-1 in Abidjan, Cote d'Ivoire, 1998-99. Bull World Health Organ 79: 641-647.

33. Nagdeo N, Thombare VR (2007) Prevention of parent-to-child transmission of HIV: an experience in rural population. Indian J Med Microbiol 25: 425.

34. Onah HE, Ibeziako N, Nkwo PO, Obi SN, Nwankwo TO (2008) Voluntary counselling and testing (VCT) uptake, nevirapine use and infant feeding options at the University of Nigeria Teaching Hospital. J Obstet Gynaecol 28: 276-279.

35. Temmerman M, Quaghebeur A, Mwanyumba F, Mandaliya K (2003) Mother-to-child HIV transmission in resource poor settings: how to improve coverage? AIDS 17: 1239-1242.

36. Shetty AK, Mhazo M, Moyo S, von LA, Mateta P, et al. (2005) The feasibility of voluntary counselling and HIV testing for pregnant women using community volunteers in Zimbabwe. Int J STD AIDS 16: 755-759.

37. Bharucha KE, Sastry J, Shrotri A, Sutar S, Joshi A, et al. (2005) Feasibility of voluntary counselling and testing services for HIV among pregnant women presenting in labour in Pune, India. Int J STD AIDS 16: 553-555.

38. Hillis SD, Rakhmanova A, Vinogradova E, Voronin E, Yakovlev A (2007) Rapid HIV testing, pregnancy, antiretroviral prophylaxis and infant abandonment in St Petersburg. Int J STD AIDS 18: 120-122.

39. Rose P, Violari A, Bolton C, Gray GE (2005) Feasibility and acceptability of postpartum voluntary counselling and testing (PPVCT) in a large tertiary hospital in the South African setting. South Afr J HIV Med: 8-10.

40. Schumacher S, Norin J, Bolu O, Dubois A, Wolford J, et al. (2009) HIV testing and counselling for prevention of mother-to-child transmission at labour and delivery in Guyana. West Indian Medical Journal 58: 112-113.

41. Pai NP, Barick R, Tulsky JP, Shivkumar PV, Cohan D, et al. (2008) Impact of round-the-clock, rapid oral fluid HIV testing of women in labor in rural India. PLoS Med 5: e92.
