# Supplementary material for: The Uptake of Integrated Perinatal Prevention of Mother-to-Child HIV Transmission Programs in Low- and Middle-Income Countries: A Systematic Review
Source: PLoS One. 2013 Mar 6;8(3):e56550. doi: 10.1371/journal.pone.0056550 (PMC3590218; doi:10.1371/journal.pone.0056550)
Supplement: Text S7 — Description of PMTCT interventions. (DOCX) [file pone.0056550.s014.docx]

**Text S7: Description of PMTCT interventions**

Various perinatal PMTCT interventions were integrated across the included studies. In 29 studies HIV counseling and testing were integrated only in antenatal care [[1-29](#_ENREF_1)], whereas in five studies they were incorporated only at labor ward [[30-34](#_ENREF_30)], and in seven studies they were integrated in both, antenatal and labor ward care [[35-41](#_ENREF_35)].

The type of HIV test used was clearly reported in 36 studies. In 27 studies rapid antibody test with a-same-day result was used [[3-5](#_ENREF_3),[7](#_ENREF_7),[8](#_ENREF_8),[10-13](#_ENREF_10),[18-20](#_ENREF_18),[22-30](#_ENREF_22),[32](#_ENREF_32),[33](#_ENREF_33),[35](#_ENREF_35),[36](#_ENREF_36),[40](#_ENREF_40),[41](#_ENREF_41)], and in 13 an additional confirmatory test was performed, either enzyme-linked immunosorbent assay (ELISA) or Western Blot [[5](#_ENREF_5),[7](#_ENREF_7),[11](#_ENREF_11),[18](#_ENREF_18),[20](#_ENREF_20),[23](#_ENREF_23),[26](#_ENREF_26),[30](#_ENREF_30),[32](#_ENREF_32),[33](#_ENREF_33),[36](#_ENREF_36),[40](#_ENREF_40),[41](#_ENREF_41)]. Six studies reported the use of either ELISA, Western Blot or enzyme immunoassay (EIA) tests with a waiting time for test results of about two weeks [[6](#_ENREF_6),[15](#_ENREF_15),[17](#_ENREF_17),[31](#_ENREF_31),[37](#_ENREF_37),[39](#_ENREF_39)]. In two studies the ELISA tests were substituted with rapid test during the study period [[1](#_ENREF_1),[21](#_ENREF_21)]. and in one study a rapid test was used at labor ward and ELISA in antenatal care [[38](#_ENREF_38)].

The opt-in strategy was implemented in 17 studies [[1-3](#_ENREF_1),[5-7](#_ENREF_5),[15](#_ENREF_15),[22-25](#_ENREF_22),[27](#_ENREF_27),[29](#_ENREF_29),[30](#_ENREF_30),[35](#_ENREF_35),[38](#_ENREF_38),[42](#_ENREF_42)], while the opt-out approach was performed in eight [[9](#_ENREF_9),[13](#_ENREF_13),[17](#_ENREF_17),[20](#_ENREF_20),[26](#_ENREF_26),[35](#_ENREF_35),[36](#_ENREF_36),[39](#_ENREF_39)]. In one study the HIV testing was mandatory [[37](#_ENREF_37)]. In the rest of the studies it was unclear what testing strategy was used.

Regimens of ARV prophylaxis varied across studies. In 24 studies women received a single dose of nevirapine in antenatal care to take at the onset of labor [[2](#_ENREF_2),[4](#_ENREF_4),[8-11](#_ENREF_8),[13](#_ENREF_13),[14](#_ENREF_14),[16-20](#_ENREF_16),[22](#_ENREF_22),[24-28](#_ENREF_24),[31](#_ENREF_31),[34-36](#_ENREF_34),[40](#_ENREF_40)]. In six studies women received zidovudine based regime during pregnancy [[1](#_ENREF_1),[7](#_ENREF_7),[15](#_ENREF_15),[21](#_ENREF_21),[23](#_ENREF_23),[39](#_ENREF_39)]. In six studies women received nevirapine or zidovudine depending on the time they were diagnosed [[3](#_ENREF_3),[5](#_ENREF_5),[12](#_ENREF_12),[30](#_ENREF_30),[37](#_ENREF_37),[38](#_ENREF_38)]. In one study prophylaxis consisted of a three drug regimen [[41](#_ENREF_41)], and in two studies of two drug regimen (zidovudine and lamivudine) provided either during pregnancy or at delivery [[6](#_ENREF_6),[32](#_ENREF_32)]. In one study women were offered zidovudine or a two drug regimen during pregnancy [[29](#_ENREF_29)]. In this study pregnant women were also provided with prophylactic highly active retroviral therapy (HAART) depending not only on the CD4 counts, but also viral load [[29](#_ENREF_29)].

ARV prophylaxis to infants was provided at labor ward except in one study where HIV positive women received nevirapine for infants at antenatal care, with an instruction to give it to their newborn at birth [[25](#_ENREF_25)]. In 26 studies infants were provided with a single dose of nevirapine [[2](#_ENREF_2),[4](#_ENREF_4),[8-11](#_ENREF_8),[13](#_ENREF_13),[14](#_ENREF_14),[16-20](#_ENREF_16),[22](#_ENREF_22),[24](#_ENREF_24),[26-28](#_ENREF_26),[31](#_ENREF_31),[33-38](#_ENREF_33),[40](#_ENREF_40)], while in six studies they received zidovudine alone or with a second drug (nevirapine or lamivudine) for a period of one to four weeks [[3](#_ENREF_3),[6](#_ENREF_6),[21](#_ENREF_21),[32](#_ENREF_32),[39](#_ENREF_39),[41](#_ENREF_41)]. In the rest of the studies the protocol did not include ARV prophylaxis for infants.

With the exception of two studies where CD4 tests and ART were provided to HIV infected women in antenatal care [[3](#_ENREF_3),[29](#_ENREF_29)], in other studies women were referred to HIV centers located in a different health facility [[21](#_ENREF_21),[35](#_ENREF_35),[36](#_ENREF_36)].

Implementation of safe delivery was reported in 10 studies [[6](#_ENREF_6),[11](#_ENREF_11),[13](#_ENREF_13),[17](#_ENREF_17),[20](#_ENREF_20),[21](#_ENREF_21),[29](#_ENREF_29),[32](#_ENREF_32),[39](#_ENREF_39),[41](#_ENREF_41)], and consisted either of avoidance of unnecessary invasive procedures during vaginal delivery or elective caesarean section.

Infants’ testing was described in 24 studies [[1](#_ENREF_1),[3](#_ENREF_3),[4](#_ENREF_4),[6](#_ENREF_6),[7](#_ENREF_7),[9](#_ENREF_9),[11-13](#_ENREF_11),[18](#_ENREF_18),[20-22](#_ENREF_20),[24](#_ENREF_24),[27-29](#_ENREF_27),[32](#_ENREF_32),[35](#_ENREF_35),[36](#_ENREF_36),[38-41](#_ENREF_38)]. This took place in various settings including maternal and child healthcare services, immunization centers, AIDS centers, other primary healthcare services or hospitals. The type of infant test and consequently the timing of it varied across studies. In 10 studies infants were tested with PCR within first six months of life [[3](#_ENREF_3),[18](#_ENREF_18),[26](#_ENREF_26),[28](#_ENREF_28),[29](#_ENREF_29),[32](#_ENREF_32),[36](#_ENREF_36),[39-41](#_ENREF_39)], and in two studies with viral load [[6](#_ENREF_6),[11](#_ENREF_11)]. In the other studies infants were tested with an HIV antibody test at 12 months or later.

Thirty one studies reported on infant feeding counseling provision [[1](#_ENREF_1),[3-5](#_ENREF_3),[7-9](#_ENREF_7),[11-13](#_ENREF_11),[15](#_ENREF_15),[17-22](#_ENREF_17),[24-29](#_ENREF_24),[32](#_ENREF_32),[33](#_ENREF_33),[35-37](#_ENREF_35),[39-41](#_ENREF_39)]. In nine of them it was performed in more than one healthcare service [[1](#_ENREF_1),[13](#_ENREF_13),[17](#_ENREF_17),[19](#_ENREF_19),[21](#_ENREF_21),[32](#_ENREF_32),[35](#_ENREF_35),[40](#_ENREF_40),[41](#_ENREF_41)]. The infant feeding recommendations varied from exclusive breastfeeding to infant formula which was provided to women for free in some studies [[1](#_ENREF_1),[3-7](#_ENREF_3),[11-13](#_ENREF_11),[15](#_ENREF_15),[17](#_ENREF_17),[21](#_ENREF_21),[22](#_ENREF_22),[29](#_ENREF_29),[36-39](#_ENREF_36)].

1. Abdullah MF, Young T, Bitalo L, Coetzee N, Myers JE (2001) Public health lessons from a pilot programme to reduce mother-to-child transmission of HIV-1 in Khayelitsha. S Afr Med J 91: 579-583.

2. Behets F, Mutombo GM, Edmonds A, Dulli L, Belting MT, et al. (2009) Reducing vertical HIV transmission in Kinshasa, Democratic Republic of Congo: trends in HIV prevalence and service delivery. AIDS Care 21: 583-590.

3. Deschamps MM, Noel F, Bonhomme J, Devieux JG, Saint-Jean G, et al. (2009) Prevention of mother-to-child transmission of HIV in Haiti. Rev Panam Salud Publica 25: 24-30.

4. Doherty TM, McCoy D, Donohue S (2005) Health system constraints to optimal coverage of the prevention of mother-to-child HIV transmission programme in South Africa: lessons from the implementation of the national pilot programme. Afr Health Sci 5: 213-218.

5. Ekouevi DK, Leroy V, Viho I, Bequet L, Horo A, et al. (2004) Acceptability and uptake of a package to prevent mother-to-child transmission using rapid HIV testing in Abidjan, Cote d'Ivoire. AIDS 18: 697-700.

6. Garcia R, Prieto F, Arenas C, Rincon J, Caicedo S, et al. (2005) [Reduction of HIV mother-to-child transmission in Colombia, two years of experience, 2003-2005]. Biomedica 25: 547-564.

7. Kanshana S, Thewanda D, Teeraratkul A, Limpakarnjanarat K, Amornwichet P, et al. (2000) Implementing short-course zidovudine to reduce mother-infant HIV transmission in a large pilot program in Thailand. AIDS 14: 1617-1623.

8. Karcher H, Kunz A, Poggensee G, Mbezi P, Mugenyi K, et al. (2006) Outcome of Different Nevirapine Administration Strategies in Preventin g Mother-to-Child Transmission (PMTCT) Programs in Tanzania and Uganda. J Int AIDS Soc 8: 12.

9. Kasenga F, Hurtig AK, Emmelin M (2007) Home deliveries: implications for adherence to nevirapine in a PMTCT programme in rural Malawi. AIDS Care 19: 646-652.

10. Kirere MM, Sondag-Thull D, Lepage P (2008) Feasibility of prevention of perinatal HIV infection by nevirapine in rural areas of the northeast Democratic Republic of Congo, 2002-2004. J Med Virol 80: 772-776.

11. Kouam L, Nsangou I, Mbanya D, Nkam M, Kongnyuy EJ, et al. (2006) Prevention of mother-to-child transmission of HIV in Cameroon: experiences from the University Teaching Hospital in Yaounde (Cameroon). Zentralbl Gynakol 128: 82-86.

12. Magoni M, Okong P, Bassani L, Kituka NP, Onyango S, et al. (2007) Implementation of a programme for the prevention of mother-to-child transmission of HIV in a Ugandan hospital over five years: challenges, improvements and lessons learned. Int J STD AIDS 18: 109-113.

13. Manzi M, Zachariah R, Teck R, Buhendwa L, Kazima J, et al. (2005) High acceptability of voluntary counselling and HIV-testing but unacceptable loss to follow up in a prevention of mother-to-child HIV transmission programme in rural Malawi: scaling-up requires a different way of acting. Trop Med Int Health 10: 1242-1250.

14. Moth IA, Ayayo ABCO, Kaseje DO (2005) Assessment of utilisation of PMTCT services at Nyanza Provincial Hospital, Kenya. SAHARA J 2: 244-250.

15. Msellati P, Hingst G, Kaba F, Viho I, Welffens-Ekra C, et al. (2001) Operational issues in preventing mother-to-child transmission of HIV-1 in Abidjan, Cote d'Ivoire, 1998-99. Bull World Health Organ 79: 641-647.

16. Nagdeo N, Thombare VR (2007) Prevention of parent-to-child transmission of HIV: an experience in rural population. Indian J Med Microbiol 25: 425.

17. Onah HE, Ibeziako N, Nkwo PO, Obi SN, Nwankwo TO (2008) Voluntary counselling and testing (VCT) uptake, nevirapine use and infant feeding options at the University of Nigeria Teaching Hospital. J Obstet Gynaecol 28: 276-279.

18. Parameshwari S, Jacob MS, Vijayakumari J, Shalini D, Sushi MK, et al. (2009) A Program on Prevention of Mother to Child Transmission of HIV at Government Hospital, Tiruchengode Taluk, Namakkal District. Indian J Community Med 34: 261-263.

19. Perez F, Mukotekwa T, Miller A, Orne-Gliemann J, Glenshaw M, et al. (2004) Implementing a rural programme of prevention of mother-to-child transmission of HIV in Zimbabwe: first 18 months of experience. Trop Med Int Health 9: 774-783.

20. Rutta E, Gongo R, Mwansasu A, Mutasingwa D, Rwegasira V, et al. (2008) Prevention of mother-to-child transmission of HIV in a refugee camp setting in Tanzania. Glob Public Health 3: 62-76.

21. Saraceni V, Rapparini C, Fonseca AF, Lima KR, Israel G, et al. Prevention of mother to child HIV transmission - a public health matter in Rio de Janeiro City; 2000; Durban, South Africa.

22. Shetty AK, Marangwanda C, Stranix-Chibanda L, Chandisarewa W, Chirapa E, et al. (2008) The feasibility of preventing mother-to-child transmission of HIV using peer counselors in Zimbabwe. AIDS Res Ther 5: 17.

23. Shetty AK, Mhazo M, Moyo S, von LA, Mateta P, et al. (2005) The feasibility of voluntary counselling and HIV testing for pregnant women using community volunteers in Zimbabwe. Int J STD AIDS 16: 755-759.

24. Stringer EM, Sinkala M, Stringer JS, Mzyece E, Makuka I, et al. (2003) Prevention of mother-to-child transmission of HIV in Africa: successes and challenges in scaling-up a nevirapine-based program in Lusaka, Zambia. AIDS 17: 1377-1382.

25. Temmerman M, Quaghebeur A, Mwanyumba F, Mandaliya K (2003) Mother-to-child HIV transmission in resource poor settings: how to improve coverage? AIDS 17: 1239-1242.

26. Torpey K, Kabaso M, Kasonde P, Dirks R, Bweupe M, et al. (2010) Increasing the uptake of prevention of mother-to-child transmission of HIV services in a resource-limited setting. BMC Health ServRes 10: 29.

27. Wanyu B, Diom E, Mitchell P, Tih PM, Meyer DJ (2007) Birth attendants trained in "Prevention of Mother-To-Child HIV Transmission" provide care in rural Cameroon, Africa. J Midwifery Womens Health 52: 334-341.

28. Welty TK, Bulterys M, Welty ER, Tih PM, Ndikintum G, et al. (2005) Integrating prevention of mother-to-child HIV transmission into routine antenatal care: the key to program expansion in Cameroon. J Acquir Immune Defic Syndr 40: 486-493.

29. Geddes R, Knight S, Reid S, Giddy J, Esterhuizen T, et al. (2008) Prevention of mother-to-child transmission of HIV programme: low vertical transmission in KwaZulu-Natal, South Africa. S Afr Med J 98: 458-462.

30. Bharucha KE, Sastry J, Shrotri A, Sutar S, Joshi A, et al. (2005) Feasibility of voluntary counselling and testing services for HIV among pregnant women presenting in labour in Pune, India. Int J STD AIDS 16: 553-555.

31. Hillis SD, Rakhmanova A, Vinogradova E, Voronin E, Yakovlev A (2007) Rapid HIV testing, pregnancy, antiretroviral prophylaxis and infant abandonment in St Petersburg. Int J STD AIDS 18: 120-122.

32. Pai NP, Barick R, Tulsky JP, Shivkumar PV, Cohan D, et al. (2008) Impact of round-the-clock, rapid oral fluid HIV testing of women in labor in rural India. PLoS Med 5: e92.

33. Rose P, Violari A, Bolton C, Gray GE (2005) Feasibility and acceptability of postpartum voluntary counselling and testing (PPVCT) in a large tertiary hospital in the South African setting. South Afr J HIV Med: 8-10.

34. Schumacher S, Norin J, Bolu O, Dubois A, Wolford J, et al. (2009) HIV testing and counselling for prevention of mother-to-child transmission at labour and delivery in Guyana. West Indian Medical Journal 58: 112-113.

35. Homsy J, Kalamya JN, Obonyo J, Ojwang J, Mugumya R, et al. (2006) Routine intrapartum HIV counseling and testing for prevention of mother-to-child transmission of HIV in a rural Ugandan hospital. J Acquir Immune Defic Syndr 42: 149-154.

36. Kissin DM, Akatova N, Rakhmanova AG, Vinogradova EN, Voronin EE, et al. (2008) Rapid HIV testing and prevention of perinatal HIV transmission in high-risk maternity hospitals in St. Petersburg, Russia. Am J Obstet Gynecol 198: 183-187.

37. Le CT, Vu TT, Luu MC, Do TN, Dinh TH, et al. (2008) Preventing mother-to-child transmission of HIV in Vietnam: an assessment of progress and future directions. J Trop Pediatr 54: 225-232.

38. Malyuta R, Newell ML, Ostergren M, Thorne C, Zhilka N (2006) Prevention of mother-to-child transmission of HIV infection: Ukraine experience to date. Eur J Public Health 16: 123-127.

39. Miranda AE, Soares RA, Prado BC, Monteiro RB, Figueiredo NC (2005) Mother to child transmission of HIV in Vitoria, Brazil: factors associated with lack of HIV prevention. AIDS Care 17: 721-728.

40. Saman M, Kruy LS, Glaziou P, Rekacewicz C, Leng C, et al. (2002) Feasibility of antenatal and late HIV testing in pregnant women in Phnom Penh Cambodia: the PERIKAM/ANRS1205 study. AIDS 16: 950-951.

41. Viani RM, Ruiz-Calderon J, Lopez G, Chacon-Cruz E, Spector SA (2010) Mother-to-child HIV transmission in a cohort of pregnant women diagnosed by rapid HIV testing at Tijuana General Hospital, Baja California, Mexico. J Int Assoc Physicians AIDS Care (ChicIll) 9: 82-86.

42. Perez F, Aung KD, Ndoro T, Engelsmann B, Dabis F (2008) Participation of traditional birth attendants in prevention of mother-to-child transmission of HIV services in two rural districts in Zimbabwe: a feasibility study. BMC Public Health 8.
