# Supplementary material for: The Uptake of Integrated Perinatal Prevention of Mother-to-Child HIV Transmission Programs in Low- and Middle-Income Countries: A Systematic Review
Source: PLoS One. 2013 Mar 6;8(3):e56550. doi: 10.1371/journal.pone.0056550 (PMC3590218; doi:10.1371/journal.pone.0056550)
Supplement: Text S8 — Data about subgroup analysis, community involvement and maternal and infant follow-up. (DOCX) [file pone.0056550.s015.docx]

**Text S8: Data about subgroup analyses, community involvement and maternal and infant follow-up**

**Impact of different strategies on uptake of PMTCT interventions integrated with antenatal care**

We analyzed the impact of opt-out and opt-in strategy on the proportion of women who were counseled, accepted to be tested and received ARV prophylaxis. Comparing the values of the five studies where opt-out testing strategy was used [[1-5](#_ENREF_1)] to the nine studies which implemented the opt-in approach [[6-14](#_ENREF_6)], we found that the testing uptake was significantly higher when opt-out testing was used: 95% (range 84-100%) versus 80% (range 26-96%), *P*=0.04. However, no significant differences were found in the proportion of women counseled; opt-out 96% (range 95-100%) versus opt-in 95% (range 30-100%), *P*=0.6034, or provided with ARV prophylaxis; opt-out 80% (range 45-99%) versus opt-in 49% (range 22-84%), *P*=0.1264.

A second subgroup analysis was performed to compare the proportion of women who received their result after being tested with rapid test compared to other types of tests. Sixteen studies provided extractable data on rapid test [[3](#_ENREF_3),[5](#_ENREF_5),[6](#_ENREF_6),[10-22](#_ENREF_10)], and two on ELISA or western blot [[4](#_ENREF_4),[9](#_ENREF_9)]. The percentage of women who received their result was significantly higher when rapid test was used: 95% (range 74-100%) versus 68% (range 67-69%) when other tests were used, *P*=0.02. However there was no significant difference in the proportion of HIV infected pregnant women who collected their results: 95% (range 73-100%) for rapid tests vs. 80% (range 60-100%) for other tests, *P*=0.71.

**Community involvement**

Several studies provided data about community mobilization. Different approaches to increasing community awareness were used in the included studies: radio or television announcements [[23-26](#_ENREF_23)], drama [[11](#_ENREF_11),[24](#_ENREF_24)], or public meetings led by peer educators [[10](#_ENREF_10)]. Community leaders (e.g. traditional, religious, refugee camp leaders) were often targeted by media campaigns [[3](#_ENREF_3),[5](#_ENREF_5),[10](#_ENREF_10),[20](#_ENREF_20)]. Traditional leaders were recruited to a program encouraging men to attend antenatal care with their partners after being reached by a campaign [[20](#_ENREF_20)]. In Zambia, male peer volunteers were recruited to promote male participation in antenatal care and reproductive health services with their partners.[[20](#_ENREF_20)] In Tanzanian refugee camp, a campaign encouraging men to attend counseling and testing with their partners was implemented [[5](#_ENREF_5)]. Male involvement was employed in several studies [[1](#_ENREF_1),[10-12](#_ENREF_10),[14](#_ENREF_14),[20](#_ENREF_20),[22](#_ENREF_22),[27](#_ENREF_27),[28](#_ENREF_28)].

**Follow-up of HIV positive women and infants after the delivery**

None of the included studies reported on the proportion of HIV positive women receiving ART after the delivery. In two studies women were referred to HIV centers for further treatment after the delivery [[29](#_ENREF_29),[30](#_ENREF_30)]. In a study from Thailand HIV positive women and their children were referred to HIV centers but the program did not include a specific HIV treatment protocol [[7](#_ENREF_7)]. Three studies reported that women with symptoms received co-trimoxazole during pregnancy or after delivery as part of the HIV care [[3](#_ENREF_3),[10](#_ENREF_10)] [[11](#_ENREF_11)].

In three articles authors stated that all identified HIV positive children were referred for HAART to HIV clinics [[17](#_ENREF_17),[31](#_ENREF_31),[32](#_ENREF_32)] Four studies reported only that HIV positive children were referred to the HIV clinics or pediatrician [[3](#_ENREF_3),[7](#_ENREF_7),[14](#_ENREF_14),[20](#_ENREF_20)].

In eight studies, infants were reportedly provided with co-trimoxazole prophylaxis but no information was provided on the number of children treated [[2](#_ENREF_2),[3](#_ENREF_3),[6](#_ENREF_6),[10](#_ENREF_10),[11](#_ENREF_11),[20](#_ENREF_20),[24](#_ENREF_24),[31](#_ENREF_31)].

1. Homsy J, Kalamya JN, Obonyo J, Ojwang J, Mugumya R, et al. (2006) Routine intrapartum HIV counseling and testing for prevention of mother-to-child transmission of HIV in a rural Ugandan hospital. J Acquir Immune Defic Syndr 42: 149-154.

2. Kasenga F, Hurtig AK, Emmelin M (2007) Home deliveries: implications for adherence to nevirapine in a PMTCT programme in rural Malawi. AIDS Care 19: 646-652.

3. Manzi M, Zachariah R, Teck R, Buhendwa L, Kazima J, et al. (2005) High acceptability of voluntary counselling and HIV-testing but unacceptable loss to follow up in a prevention of mother-to-child HIV transmission programme in rural Malawi: scaling-up requires a different way of acting. Trop Med Int Health 10: 1242-1250.

4. Onah HE, Ibeziako N, Nkwo PO, Obi SN, Nwankwo TO (2008) Voluntary counselling and testing (VCT) uptake, nevirapine use and infant feeding options at the University of Nigeria Teaching Hospital. J Obstet Gynaecol 28: 276-279.

5. Rutta E, Gongo R, Mwansasu A, Mutasingwa D, Rwegasira V, et al. (2008) Prevention of mother-to-child transmission of HIV in a refugee camp setting in Tanzania. Glob Public Health 3: 62-76.

6. Abdullah MF, Young T, Bitalo L, Coetzee N, Myers JE (2001) Public health lessons from a pilot programme to reduce mother-to-child transmission of HIV-1 in Khayelitsha. S Afr Med J 91: 579-583.

7. Kanshana S, Thewanda D, Teeraratkul A, Limpakarnjanarat K, Amornwichet P, et al. (2000) Implementing short-course zidovudine to reduce mother-infant HIV transmission in a large pilot program in Thailand. AIDS 14: 1617-1623.

8. Malyuta R, Newell ML, Ostergren M, Thorne C, Zhilka N (2006) Prevention of mother-to-child transmission of HIV infection: Ukraine experience to date. Eur J Public Health 16: 123-127.

9. Msellati P, Hingst G, Kaba F, Viho I, Welffens-Ekra C, et al. (2001) Operational issues in preventing mother-to-child transmission of HIV-1 in Abidjan, Cote d'Ivoire, 1998-99. Bull World Health Organ 79: 641-647.

10. Perez F, Mukotekwa T, Miller A, Orne-Gliemann J, Glenshaw M, et al. (2004) Implementing a rural programme of prevention of mother-to-child transmission of HIV in Zimbabwe: first 18 months of experience. Trop Med Int Health 9: 774-783.

11. Shetty AK, Marangwanda C, Stranix-Chibanda L, Chandisarewa W, Chirapa E, et al. (2008) The feasibility of preventing mother-to-child transmission of HIV using peer counselors in Zimbabwe. AIDS Res Ther 5: 17.

12. Shetty AK, Mhazo M, Moyo S, von LA, Mateta P, et al. (2005) The feasibility of voluntary counselling and HIV testing for pregnant women using community volunteers in Zimbabwe. Int J STD AIDS 16: 755-759.

13. Temmerman M, Quaghebeur A, Mwanyumba F, Mandaliya K (2003) Mother-to-child HIV transmission in resource poor settings: how to improve coverage? AIDS 17: 1239-1242.

14. Geddes R, Knight S, Reid S, Giddy J, Esterhuizen T, et al. (2008) Prevention of mother-to-child transmission of HIV programme: low vertical transmission in KwaZulu-Natal, South Africa. S Afr Med J 98: 458-462.

15. Doherty TM, McCoy D, Donohue S (2005) Health system constraints to optimal coverage of the prevention of mother-to-child HIV transmission programme in South Africa: lessons from the implementation of the national pilot programme. Afr Health Sci 5: 213-218.

16. Ekouevi DK, Leroy V, Viho I, Bequet L, Horo A, et al. (2004) Acceptability and uptake of a package to prevent mother-to-child transmission using rapid HIV testing in Abidjan, Cote d'Ivoire. AIDS 18: 697-700.

17. Kouam L, Nsangou I, Mbanya D, Nkam M, Kongnyuy EJ, et al. (2006) Prevention of mother-to-child transmission of HIV in Cameroon: experiences from the University Teaching Hospital in Yaounde (Cameroon). Zentralbl Gynakol 128: 82-86.

18. Saman M, Kruy LS, Glaziou P, Rekacewicz C, Leng C, et al. (2002) Feasibility of antenatal and late HIV testing in pregnant women in Phnom Penh Cambodia: the PERIKAM/ANRS1205 study. AIDS 16: 950-951.

19. Stringer EM, Sinkala M, Stringer JS, Mzyece E, Makuka I, et al. (2003) Prevention of mother-to-child transmission of HIV in Africa: successes and challenges in scaling-up a nevirapine-based program in Lusaka, Zambia. AIDS 17: 1377-1382.

20. Torpey K, Kabaso M, Kasonde P, Dirks R, Bweupe M, et al. (2010) Increasing the uptake of prevention of mother-to-child transmission of HIV services in a resource-limited setting. BMC Health ServRes 10: 29.

21. Wanyu B, Diom E, Mitchell P, Tih PM, Meyer DJ (2007) Birth attendants trained in "Prevention of Mother-To-Child HIV Transmission" provide care in rural Cameroon, Africa. J Midwifery Womens Health 52: 334-341.

22. Welty TK, Bulterys M, Welty ER, Tih PM, Ndikintum G, et al. (2005) Integrating prevention of mother-to-child HIV transmission into routine antenatal care: the key to program expansion in Cameroon. J Acquir Immune Defic Syndr 40: 486-493.

23. Garcia R, Prieto F, Arenas C, Rincon J, Caicedo S, et al. (2005) [Reduction of HIV mother-to-child transmission in Colombia, two years of experience, 2003-2005]. Biomedica 25: 547-564.

24. Karcher H, Kunz A, Poggensee G, Mbezi P, Mugenyi K, et al. (2006) Outcome of Different Nevirapine Administration Strategies in Preventin g Mother-to-Child Transmission (PMTCT) Programs in Tanzania and Uganda. J Int AIDS Soc 8: 12.

25. Le CT, Vu TT, Luu MC, Do TN, Dinh TH, et al. (2008) Preventing mother-to-child transmission of HIV in Vietnam: an assessment of progress and future directions. J Trop Pediatr 54: 225-232.

26. Moth IA, Ayayo ABCO, Kaseje DO (2005) Assessment of utilisation of PMTCT services at Nyanza Provincial Hospital, Kenya. SAHARA J 2: 244-250.

27. Kirere MM, Sondag-Thull D, Lepage P (2008) Feasibility of prevention of perinatal HIV infection by nevirapine in rural areas of the northeast Democratic Republic of Congo, 2002-2004. J Med Virol 80: 772-776.

28. Magoni M, Okong P, Bassani L, Kituka NP, Onyango S, et al. (2007) Implementation of a programme for the prevention of mother-to-child transmission of HIV in a Ugandan hospital over five years: challenges, improvements and lessons learned. Int J STD AIDS 18: 109-113.

29. Kissin DM, Akatova N, Rakhmanova AG, Vinogradova EN, Voronin EE, et al. (2008) Rapid HIV testing and prevention of perinatal HIV transmission in high-risk maternity hospitals in St. Petersburg, Russia. Am J Obstet Gynecol 198: 183-187.

30. Saraceni V, Rapparini C, Fonseca AF, Lima KR, Israel G, et al. Prevention of mother to child HIV transmission - a public health matter in Rio de Janeiro City; 2000; Durban, South Africa.

31. Deschamps MM, Noel F, Bonhomme J, Devieux JG, Saint-Jean G, et al. (2009) Prevention of mother-to-child transmission of HIV in Haiti. Rev Panam Salud Publica 25: 24-30.

32. Viani RM, Ruiz-Calderon J, Lopez G, Chacon-Cruz E, Spector SA (2010) Mother-to-child HIV transmission in a cohort of pregnant women diagnosed by rapid HIV testing at Tijuana General Hospital, Baja California, Mexico. J Int Assoc Physicians AIDS Care (ChicIll) 9: 82-86.
